# Pharmacologic inhibition of STAT5 in acute myeloid leukemia

**DOI:** 10.1038/s41375-017-0005-9

**Published:** 2018-02-02

**Authors:** Bettina Wingelhofer, Barbara Maurer, Elizabeth C. Heyes, Abbarna A. Cumaraswamy, Angelika Berger-Becvar, Elvin D. de Araujo, Anna Orlova, Patricia Freund, Frank Ruge, Jisung Park, Gary Tin, Siawash Ahmar, Charles-Hugues Lardeau, Irina Sadovnik, Dávid Bajusz, György Miklós Keserű, Florian Grebien, Stefan Kubicek, Peter Valent, Patrick T. Gunning, Richard Moriggl

**Affiliations:** 1 Ludwig Boltzmann Institute for Cancer Research, Vienna, Austria; 2 Institute of Animal Breeding and Genetics, University of Veterinary Medicine, Vienna, Austria; 3 Institute of Pharmacology and Toxicology, University of Veterinary Medicine, Vienna, Austria; 4 Department of Chemical & Physical Sciences, University of Toronto Mississauga, Mississauga, ON Canada; 5 Department of Chemistry, University of Toronto, Toronto, ON Canada; 6 Research Center for Molecular Medicine (CeMM), Vienna, Austria; 7 Department of Internal Medicine I, Division of Hematology & Hemostaseology, Ludwig Boltzmann Cluster Oncology, Medical University of Vienna, Vienna, Austria; 8 Research Centre for Natural Sciences, Hungarian Academy of Sciences, Budapest, Hungary; 9 Medical University of Vienna, Vienna, Austria

**Keywords:** Drug development, Targeted therapies, Leukaemia

## Abstract

The transcription factor STAT5 is an essential downstream mediator of many tyrosine kinases (TKs), particularly in hematopoietic cancers. STAT5 is activated by FLT3-ITD, which is a constitutively active TK driving the pathogenesis of acute myeloid leukemia (AML). Since STAT5 is a critical mediator of diverse malignant properties of AML cells, direct targeting of STAT5 is of significant clinical value. Here, we describe the development and preclinical evaluation of a novel, potent STAT5 SH2 domain inhibitor, AC-4–130, which can efficiently block pathological levels of STAT5 activity in AML. AC-4–130 directly binds to STAT5 and disrupts STAT5 activation, dimerization, nuclear translocation, and STAT5-dependent gene transcription. Notably, AC-4–130 substantially impaired the proliferation and clonogenic growth of human AML cell lines and primary FLT3-ITD^+^ AML patient cells in vitro and in vivo. Furthermore, AC-4–130 synergistically increased the cytotoxicity of the JAK1/2 inhibitor Ruxolitinib and the p300/pCAF inhibitor Garcinol. Overall, the synergistic effects of AC-4–130 with TK inhibitors (TKIs) as well as emerging treatment strategies provide new therapeutic opportunities for leukemia and potentially other cancers.

## Introduction

STAT5 is a key member of the JAK/STAT core cancer pathway, activated by a plethora of cytokines and growth factors to regulate a wide spectrum of physiologic processes in hematopoietic cells [1, 2]. Persistent STAT5 activity (pY-STAT5) is found in many hematopoietic cancers driven by hyper-activated upstream TKs, where it is essential for leukemia cell maintenance and survival [3–6]. High pY-STAT5 levels have been implicated as a negative prognostic marker in myeloid malignancies [7] and have been associated with tyrosine kinase inhibitor (TKI)-resistance [8].

Acute myeloid leukemia (AML) is one of the most common blood cancers in adults, with the majority of patients being over 60 years old. Despite considerable advances in therapeutic approaches and allogeneic hematopoietic stem cell transplantation, patient outcomes remain poor [9]. Activating mutations in the FLT3 receptor TK represent the most frequent mutations in AML, affecting 28% of all patients [10, 11]. The most common class of *FLT3-*mutations are internal tandem duplications (ITDs) [12]. FLT3-ITD mutations lead to constitutive, ligand-independent activation of the kinase [13], and persistent activation of downstream signaling pathways involving PI3K-AKT, RAS-MAPK, and STAT5.

So far, many attempts have been made to achieve pharmacological inhibition of STAT signaling in AML, mainly using TKIs that specifically target FLT3. However, cancer cells frequently develop resistance to these drugs, often via mutations that facilitate continued activation of STATs. Because of the critical role of STAT5 in mediating the effects of various mutated TKs, its direct targeting may be an effective alternative anti-cancer strategy. Direct inhibitors of STAT family members are currently in different stages of pharmaceutical drug development [14–18]. However, many of these compounds have either an indirect effect on JAK/STAT signaling or target specificity was unverified. More recently, STAT5-specific inhibitors such as Stafib-2, a bisphosphate-containing small molecule [19], or AC-3–19, a salicylic acid-based STAT5 SH2 domain inhibitor [20], have been developed but were not potent enough for clinical translation.

Here, we describe a novel potent chemical probe that specifically targets the STAT5 SH2 domain. Our lead compound AC-4–130 binds to and efficiently blocks STAT5 activation and subsequent transcriptional activity. AC-4–130 showed high cytotoxic potential in FLT3-ITD^+^ AML cells in vitro and in vivo. These effects were enhanced when AC-4–130 was combined with the JAK1/2 inhibitor Ruxolitinib or the p300/pCAF inhibitor Garcinol. Together, our results implicate AC-4–130 as an effective strategy to target AML cell growth and survival.

## Materials and methods

### RNA-seq processing, analysis, and gene set enrichment analysis (GSEA)

MV4–11 and MOLM-13 cells were treated in triplicate with 5 µM AC-4–130 or dimethyl sulfoxide (DMSO) (Ctrl) for 24 h followed by total RNA extraction using the RNeasy Mini Kit (Qiagen, Venlo, Netherlands). RNA-seq 50 bp single-end libraries were sequenced on a HiSeq 2000 (Illumina, San Diego, CA, USA), resulting in average 31.4 M reads per replicate. Detailed bioinformatics analysis is described in the Supplementary Methods. The data have been deposited to GEO (GSE103510).

### High-throughput synergy screening

Compounds in 50 nl DMSO were plated onto 384-well plates at 5000-fold their respective maximum plasma concentration in humans. Additionally, AC-4–130 (2 µM) or DMSO was added. MV4–11 and MOLM-13 cells (5000 cells/well) were seeded with the compounds, incubated for 72 h, and cell viability was measured with CellTiterGlo (Promega, Madison, WI, USA). Data was normalized to a negative control (DMSO; 100% viability) and positive control (1 µM Bortezomib; 0% viability). Hits were defined based on the following criteria: DMSO controls >30%, Viability difference >50%. Compound synergy was determined as previously described [21] using Isobologram analysis [22].

### Statistical analysis

Statistical calculations were performed using GraphPad Prism 5.01 software and data are reported as mean ± SEM. Experiments were performed in triplicates and/or repeated at least three times unless indicated otherwise. Two-tailed Student’s *t*-tests and Wilcoxon rank-sum tests were used for comparing two groups and one-way ANOVA followed by Tukey’s honestly significant difference, Dunn’s multiple comparison or Bonferroni post hoc tests were used for comparing multiple groups. In case of non-equal variances, Welch’s correction was performed. *p*-values are considered as follows: * *p*-value < 0.05; ** *p*-value < 0.01; and *** *p*-value < 0.001.

Additional Materials and methods are described in Supplementary Methods.

## Results

### AC-4–130 is a potent STAT5 inhibitor

**Fig. 1:**
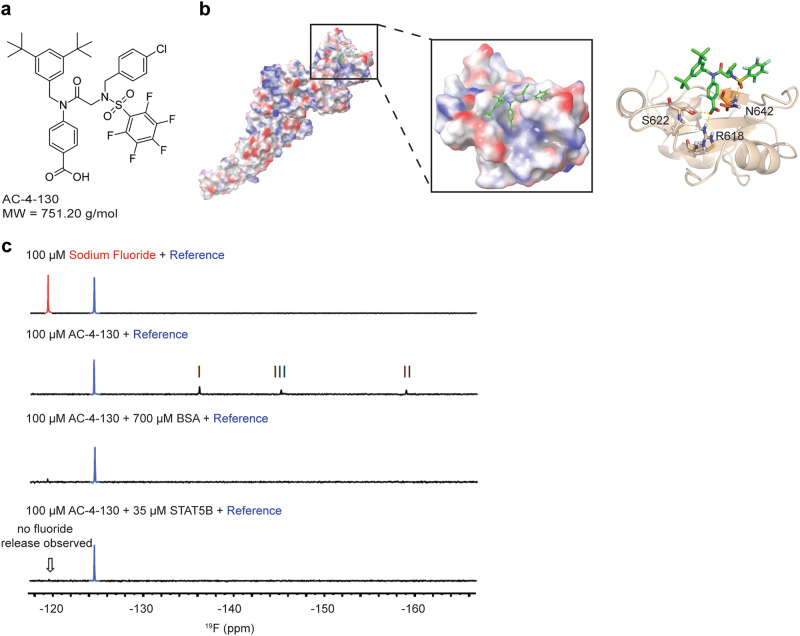
In vitro characterization of AC-4–130. **a** Chemical structure of AC-4–130. **b** Schematic representation of the STAT5B domain structure and binding mode of AC-4–130. **c** 1D ^19^F NMR studies of AC-4–130 with STAT5B

Previously, we identified a class of salicylic acid-containing small molecules that bind to STAT3 with high selectivity over STAT1 and STAT5 (Stat3 *K*_i_ = 15 μM; Stat5, *K*_i_ > 25 μM; Stat1, *K*_i_ > 25 μM) [23–26]. Since the SH2 domains of STAT5A/B share only ~40% homology with STAT3 or STAT1 (Supplementary Fig. 1a), we hypothesized that this would allow for the development of selective STAT5 inhibitors. Biophysical screening of a library of SH2 domain binders that show high selectivity for STAT5 over STAT1 and STAT3 [20] identified the STAT5-binding small molecule AC-4–130 (Fig. 1a).

To verify if AC-4–130 binds to the SH2 domain, a thermal shift assay with full-length STAT5B was performed using a fluorescently labeled EPO receptor peptide which interacts with the SH2 domain. In the absence of any inhibitor, the peptide was shown to bind STAT5B (Supplementary Fig 1b). The presence of AC-4–130 effectively displaced the peptide from the STAT5B protein, indicating binding of AC-4–130 to the STAT5 SH2 domain.

Next, the binding mode of AC-4–130 was evaluated by docking the compound to the modeled structure of the STAT5B SH2 domain (Fig. 1b). Docking calculations revealed that the benzoic acid moiety of AC-4–130 binds into the SH2 domain and interacts with residues that are involved in pY binding [27], specifically Arg-618 and Ser-622 of the conserved pY-binding FLLRFSDS motif. The 4-chlorobenzyl and pentafluorobenzene sulfonamide (PFBS) groups of AC-4–130 are engaged in *π*–*π* contacts with two adjacent amphiphilic pockets providing target affinity. Furthermore, a benzyl moiety participates in a cation–*π* interaction with Asn-642 that is unique to the STAT5 SH2 domain compared to STAT1 and STAT3.

Importantly, the reactivity of the para-position of the PFBS to thiol-based nucleophiles, including those found on STAT proteins [25], allowed for 1D ^19^F nuclear magnetic resonance (NMR) studies. Initially, binding of a reported covalent STAT3 inhibitor to STAT3 was tested to validate the binding assay (Supplementary Fig. 1c). Binding of SH4–54 to STAT3 resulted in the disappearance of fluorine peaks, representing the PFBS group of the compound. Concomitantly, free fluorine, the by-product of a protein-PFBS covalent reaction, was detected in solution indicating covalent binding to STAT3. When we incubated STAT5B with AC-4–130, the fluorine peaks of the PFBS group again disappeared upon binding of the inhibitor to the protein. However, fluorine ion production was not observed indicating a non-covalent interaction (Fig. 1c). These experiments collectively demonstrate that AC-4–130 targets the SH2 domain of the STAT5 protein.

### AC-4–130 disrupts STAT5 dimerization and transcriptional activity

**Fig. 2:**
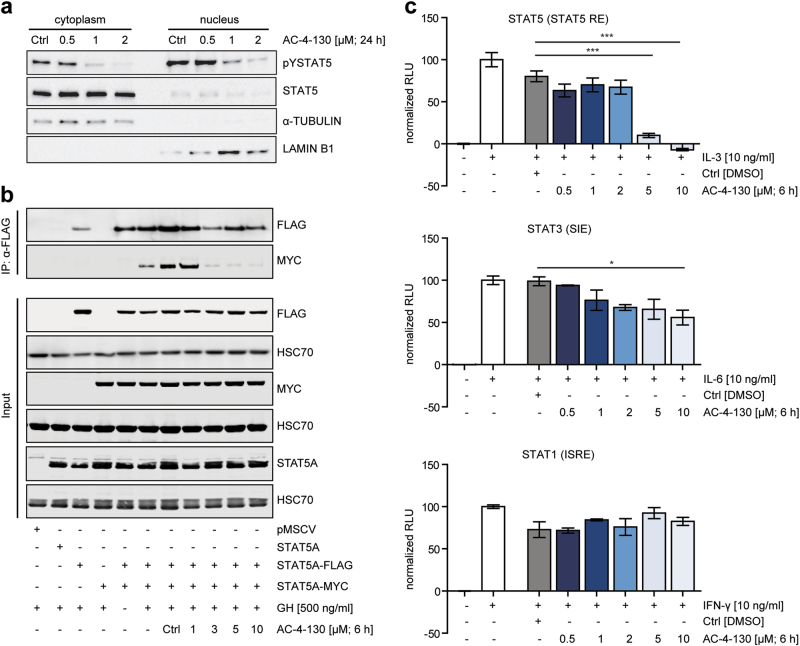
AC-4–130 inhibits STAT5 dimerization and target gene expression. **a** Subcellular fractions of Ba/F3 FLT3-ITD^+^ cells immunoblotted for pY-STAT5 and total STAT5. α-TUBULIN and LAMIN B1 were used as loading controls for cytoplasmic and nuclear fractions, respectively. Blots represent 2 independent experiments. Uncropped version of the Western blot is shown in Supplementary Fig. 8. **b** STAT5A-MYC and STAT5A-FLAG were co-transfected into HEK293T cells, co-immunoprecipitated with anti-FLAG and blotted with anti-FLAG and anti-MYC. Whole cell lysates were immunoblotted for MYC- or FLAG-tag, STAT5A, and HSC70 to show input. Results represent two independent experiments. Uncropped version of the Western blot is shown in Supplementary Fig. 8. **c** Ba/F3 cells were electroporated with Luciferase (*Firefly*) reporter plasmid for STAT5, and HT-29 cells were transfected with reporter plasmids for STAT1 or STAT3 in addition to pRL-TK (*Renilla* luciferase). Cells were starved, pretreated with AC-4–130 or DMSO (Ctrl) for 6 h and stimulation with appropriate cytokine. Relative luciferase activity was determined using the Dual-Luciferase Reporter Assay

Next, we investigated the cellular activity of AC-4–130 in the context of variable STAT5 expression or activity modeled by different Ba/F3 cells lines [28–31] (Supplementary Fig. 2a). We used the constitutive active STAT5 variants cS5^F^ and cS5^RF^, which were shown to be persistently active in the absence of exogenous cytokine stimuli and promote myeloid hyperplasia in murine transplantation models [28]. Furthermore, we established cell lines overexpressing either wild type (wt) STAT5B or STAT5B^N642H^, a frequent recurrent hotspot mutation in various types of aggressive T-cell neoplasias [29–31]. Concerning cell survival, parental Ba/F3 cells were the most sensitive and Ba/F3 STAT5B^N642H^ cells the most resistant towards AC-4–130 (Supplementary Fig. 2b). Interestingly, we found a direct correlation between survival and pY-STAT5 levels (Supplementary Fig. 2c). Treatment of the Ba/F3 cell lines with AC-4–130 decreased pY-STAT5 levels in parental, cS5^RF^- and STAT5B-overexpressing cells (Supplementary Fig. 2d). However, AC-4–130 induced only a minor decrease in cS5^F^ and STAT5B^N642H^ expressing cells at the highest concentrations. HT-29 cells treated with IL-6 or IFN-γ to stimulate pY-STAT3 or pY-STAT1, respectively, were mainly unaffected by AC-4–130 (Supplementary Fig. 2e). Analysis of the subcellular localization of pY-STAT5 and STAT5 upon AC-4–130 treatment revealed reduced pY-STAT5 levels both in the cytoplasm and nucleus, as well as reduced overall levels of nuclear STAT5 (Fig. 2a, Supplementary Fig. 2f). Next, we tested whether AC-4–130 would disrupt the dimerization of STAT5. HEK293T cells co-transfected with STAT5A-FLAG and STAT5A-MYC were treated with AC-4–130 before stimulation with growth hormone (GH). GH receptor stimulation induced parallel pY-STAT5 dimerization of FLAG and MYC-tagged STAT5A, which was efficiently inhibited by AC-4–130 (Fig. 2b). Finally, AC-4–130 blocked the ability of pY-STAT5 to activate a β-casein-luciferase reporter construct, while the transcriptional activities of pY-STAT3 and pY-STAT1 were largely unaffected (Fig. 2c). These results indicate that AC-4–130 effectively blocks events associated with STAT5 activity, including phosphorylation, dimerization, nuclear translocation, and transcriptional activity.

### FLT3-ITD^+^ AML cells are most susceptible to STAT5 inhibition

**Fig. 3:**
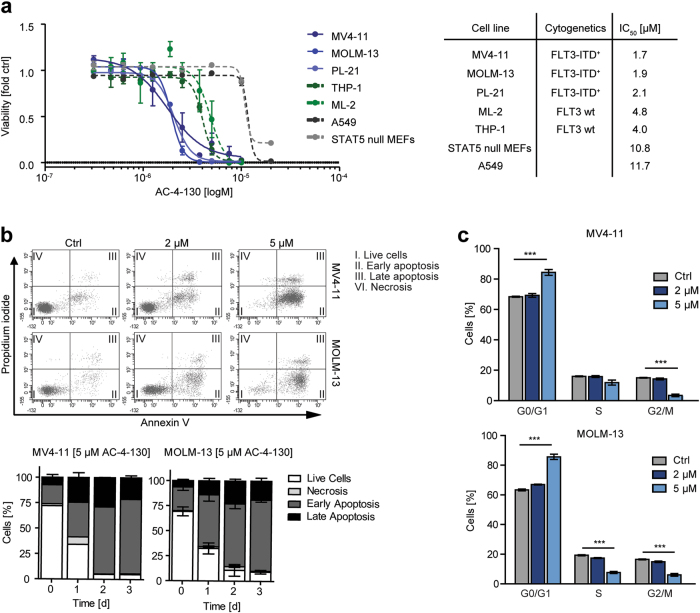
FLT3-ITD^+^ cells are most susceptible to AC-4–130. **a** Viability assay for hematopoietic or control cell lines with AC-4–130 or DMSO (Ctrl) for 72 h. IC_50_ values (µM) were determined using GraphPad Prism 5 software (GraphPad Software, Inc.). **b** MV4–11 and MOLM-13 cells were treated with AC-4–130 or DMSO (Ctrl) in a dose-dependent manner for 72 h or with 5 µM AC-4–130 in a time-dependent manner. Apoptotic cells were detected by AnnexinV/PI staining. Representative dot plots are shown. **c** Cell cycle distribution was determined after 72 h using PI staining

To obtain insight into the consequences of STAT5 inhibition in human FLT-ITD^+^ AML cells, we exposed cell lines harboring either wt FLT3 or FLT3-ITD to AC-4–130. Interestingly. FLT3-ITD^+^ cells were more sensitive to STAT5 inhibition (Fig. 3a). Treatment of MV4–11 or MOLM-13 cells led to a significant increase in apoptosis in a dose-dependent and time-dependent manner (Fig. 3b, Supplementary Fig. 3a), accompanied by cleavage of Caspase 3 and poly (ADP-ribose) polymerase (Supplementary Fig. 3b), and elevated levels of active Caspase 3/7 (Supplementary Fig. 3c). Furthermore, AC-4–130 induced cell cycle arrest with an increase in G0/G1 arrested cells and a concomitant reduction in cells in S or G2/M (Fig. 3c, Supplementary Fig. 3d). Additionally, AC-4–130 reduced the clonogenic potential of MV4–11 and MOLM-13 cells in semi-solid medium (Supplementary Fig. 3e). Taken together, AC-4–130 inhibits the proliferation and clonogenic growth of FLT3-ITD-driven leukemic cells by inducing cell cycle arrest and apoptosis.

### AC-4–130 suppresses STAT5 target gene expression

**Fig. 4:**
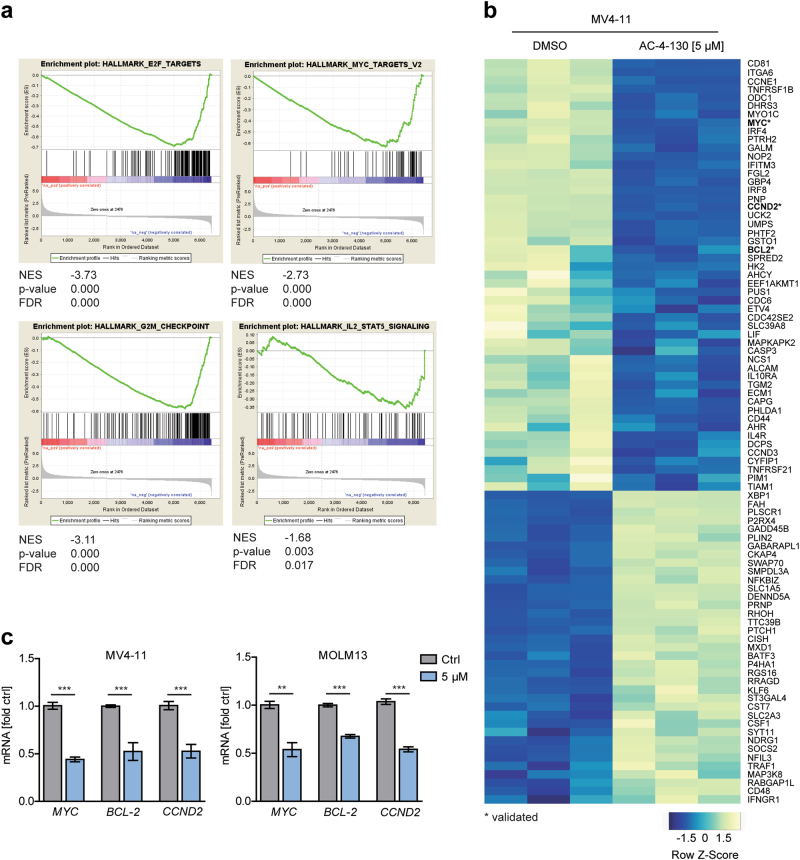
RNA-seq analysis shows downregulation of the IL-2 STAT5 pathway. **a** GSEA of differentially expressed genes (*p*-value ≤ 0.01) in MV4–11 cells treated with AC-4–130 (5 µM) or DMSO (Ctrl) for 24 h. **b** Heatmap of differentially expressed genes in MV4–11 cells enriched in the IL-2 STAT5 hallmark pathway. **c** MV4–11 and MOLM-13 cells were treated with AC-4–130 or DMSO (Ctrl) for 24 h. mRNA expression of STAT5 target genes was analyzed by RT-qPCR. Data were normalized to *GAPDH*

To identify AC-4–130-induced global changes in gene expression patterns we performed RNA-seq using FLT3-ITD^+^ cell lines MV4–11 and MOLM-13 after inhibitor treatment. We found a significant downregulation of 1418 genes and upregulation of 752 genes in both cell lines (Supplementary Fig. 4a). Importantly, GSEA confirmed that downregulated genes were significantly enriched in the hallmark IL-2-STAT5 signaling pathway. Additionally, gene sets representative of E2F targets, G2M checkpoint and MYC targets were also downregulated upon inhibitor treatment, substantiating the fact that STAT5 is a driver of cell-cycle progression (Fig. 4a, Supplementary Fig. 4b). Well-described STAT5 target genes including *MYC*, *CCND2*, and *BCL2* were among the top downregulated genes, verifying targeted inhibition of STAT5 (Fig. 4b,c, Supplementary Fig. 4c). Overall, AC-4–130 treatment led to decreased expression of STAT5 target genes that are essential to AML progression.

### AC-4–130 impairs clonogenic growth of human AML cancer stem cells

**Fig. 5:**
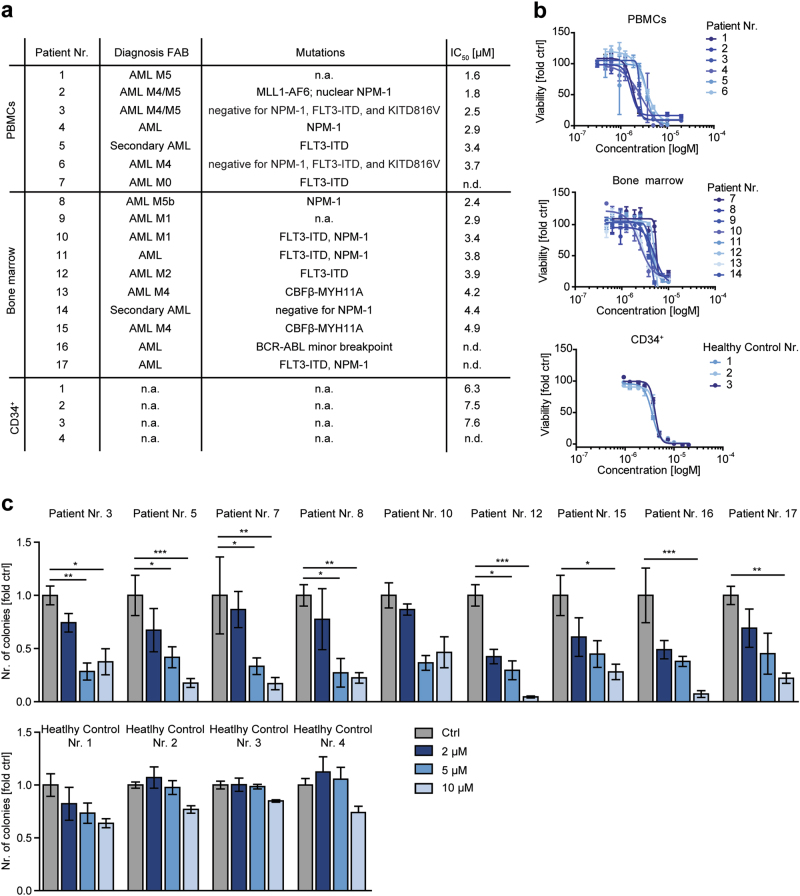
AC-4–130 reduces clonogenicity of primary AML patient cancer stem cells. **a** Characteristics of human AML patients. **b** Viability assay of human AML patient samples and healthy CD34^+^ cells treated with AC-4–130 or DMSO (Ctrl) for 48 h. **c** AML samples and CD34^+^ cells were embedded in methylcellulose in the presence of AC-4–130 or DMSO (Ctrl). Colonies were counted 10 days after seeding

To test the potential of AC-4–130 for clinical translation, we tested 14 primary human AML samples with different, frequently occurring driver mutations (Fig. 5a) for their response to STAT5 inhibition. AC-4–130 significantly reduced the viability of all samples (Fig. 5a, b, Supplementary Fig. 5a and 5b), substantiating the central role of STAT5 in the maintenance of AML cells. Interestingly, the sensitivity of patient cells to AC-4–130 correlated with the amount of CD34^+^/CD38^−^ cells in the respective sample (Supplementary Fig. 5c). Importantly, healthy CD34^+^ cells isolated from umbilical cord blood were less sensitive towards AC-4–130 (Fig. 5a, b, Supplementary Fig. 5a and 5b). Next, we analyzed the effect of AC-4–130 on the clonogenic growth of AML patient samples. AC-4–130 decreased the number of colonies of all AML samples while the clonogenic growth of healthy CD34^+^ cells was largely unaffected (Fig. 5c, Supplementary Fig. 5d). Prolonged exposure of cells to increasing concentrations of AC-4–130 induced apoptosis (Supplementary Fig. 5e). Thus, AC-4–130-mediated STAT5 inhibition efficiently blocks the proliferation and clonogenic growth of primary human AML cells, while healthy CD34^+^ cells are less sensitive.

### AC-4–130 treatment blocks *FLT3*-ITD driven cancer cell growth in vivo

To investigate the in vivo activity of AC-4–130, we treated C57BL/6J mice with AC-4–130 (25 mg/kg) or vehicle for 21 days by daily intraperitoneal injection. No significant loss of body weight or defects in hematopoiesis (white blood cell count and hematocrit) was observed (Supplementary Fig. 6a and 6b). Furthermore, granulocyte, T-cell and B-cell numbers in blood, bone marrow, and lymph nodes, as well as stem cell numbers in the bone marrow were largely normal (Supplementary Fig. 6c). Parameters of acute liver and kidney damage were not enhanced (Supplementary Fig. 6d). Thus, we conclude that AC-4–130 is well tolerated and does not induce hematopoietic defects in wt mice.

**Fig. 6:**
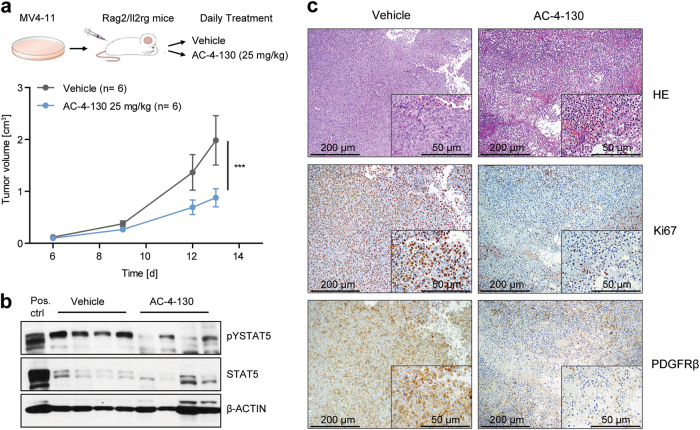
AC-4–130 decreases tumor formation and leukemogenesis in vivo. **a** Tumor volume of MV4–11 cells subcutaneously injected into both flanks of Rag2^−/−^γc^−/−^ recipients, treated daily with vehicle or AC-4–130 (25 mg/kg). **b** Immunoblot showing pY-STAT5 and STAT5 levels after treatment. β-ACTIN was used as loading control. Uncropped version of the Western blot is shown in Supplementary Fig. 9. **c** H&E staining, Ki67, and PDGFRβ immunohistochemical staining of subcutaneously grown tumors

To assess whether AC-4–130 could inhibit STAT5 activity in an in vivo leukemia setting, we used a FLT3-ITD–dependent MV4–11 tumor xenograft model. Cells were subcutaneously implanted into Rag2^−/−^γc^−/−^ mice and AC-4–130 was administered daily. AC-4–130 treatment resulted in a clear reduction in tumor growth (Fig. 6a), tumor volume (Supplementary Fig. 6e), and STAT5 activity (Fig. 6b, Supplementary Fig. 6f). This was accompanied by a profound reduction in Ki67 and PDGFRβ staining (Fig. 6c) indicating decreased proliferation and vascularization. These experiments show that AC-4–130 is able to inhibit the proliferation of AML cells in vivo.

### AC-4–130 sensitizes AML cells to TK inhibition

**Fig. 7:**
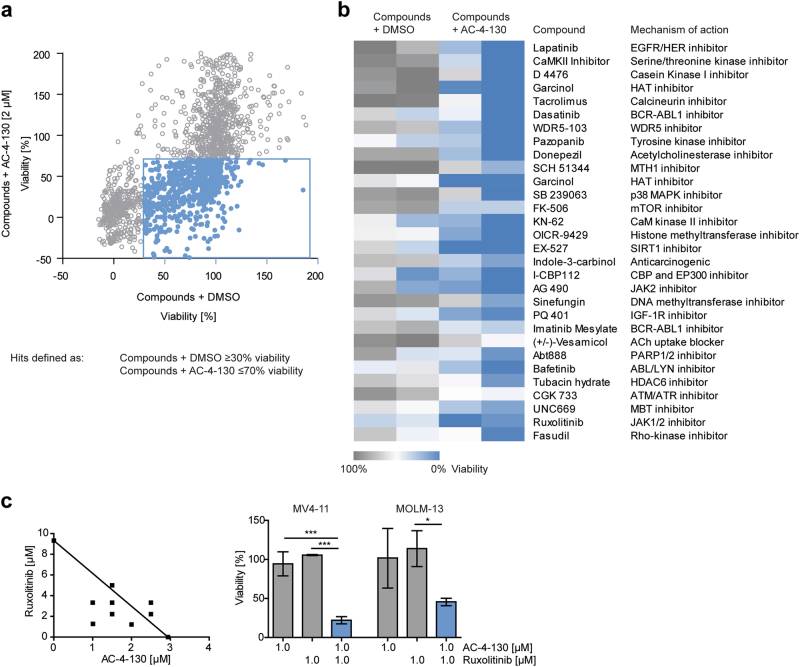
A chemical screen reveals compounds acting synergistically with AC-4–130. **a** MOLM-13 and MV4–11 cells were treated with a library of FDA-approved and experimental drugs (10–50 µM) alone or in combination with AC-4–130 (2 µM) for 72 h and cell viability was assessed. **b** Heatmap of hits defined as compounds giving a viability difference of 50% compared to DMSO controls. **c** Cell viability of MV4–11 and MOLM-13 cells treated with single drugs or combinations for 24 h. Synergy was assessed using Isobolograms

The genetic heterogeneity of AML demands various treatment options to guarantee optimal management of individual patients. Due to the existence of several mutations in any given cancer cell, it is unlikely that any single agent will induce long-lasting remission. To identify additive or synergistic effects, we screened a library of >1800 compounds including U.S. FDA-approved drugs and investigational compounds, in combination with AC-4–130 (Fig. 7a). After exclusion of agents with no implications for cancer treatment, 30 promising combinations remained for further validation (Fig. 7b). The combination of AC-4–130 and the JAK1/2 inhibitor Ruxolitinib or the p300/pCAF inhibitor Garcinol revealed a pronounced synergistic effect in MV4–11 and MOLM-13 cells (Fig. 7c, Supplementary Fig. 7a). Results were confirmed using the JAK2 inhibitor AG490 and the CBP/p300 inhibitor I-CBP112 (Supplementary Fig. 7a and 7b). Overall, these data demonstrate that the synergistic effects of AC-4–130-mediated STAT5 inhibition together with the inhibition of other pathways are of potential therapeutic value and reveal new mechanistic insights into the role STAT5 signaling in AML.

## Discussion

The need for specific compounds targeting STAT5 is highlighted by the well-established role of STAT5 in hematopoietic cancers [3–6]. Here, we developed and extensively characterized the novel SH2 domain-targeting STAT5 inhibitor AC-4–130. Moreover, we demonstrated FLT3-ITD^+^ AML cell sensitivity to AC-4–130 treatment in vitro and in vivo. Thus, we propose that AC-4–130 is not only an excellent tool compound but also an interesting lead structure that, upon further development, could achieve potential clinical relevance for targeting STAT5-driven hematopoietic diseases.

Drug screening and structure-based design focusing on targeting the SH2 domain has led to the discovery of several STAT5 inhibitors, e.g., Pimozide, with encouraging in vitro activities [15, 17, 18]. However, most of these studies failed to prove target specificity and selectivity. In contrast to other STAT5 inhibitors, AC-4–130 has chemical properties essential for a valuable chemical probe. ^19^F NMR binding experiments, thermoshift assays and in silico modeling showed on-target binding of AC-4–130 through non-covalent interactions of several side groups with conserved residues of the STAT5 SH2 domain. Furthermore, AC-4–130 specifically targets cellular STAT5 at pharmacologically relevant concentrations while having little effect on STAT3 and STAT1. As such, AC-4–130 represents an excellent tool to clarify important canonical functions of pY-STAT5, as well as non-canonical functions like the role of unphosphorylated STAT5 (uSTAT5) [32, 33] in chromatin organization.

FLT3-ITD^+^ AML cell lines were most sensitive to STAT5 inhibition, emphasizing the importance of STAT5 in the maintenance and survival of cells with this particular driver mutation [12]. Our findings are supported by the analysis of primary AML samples, where AC-4–130 caused a significant decrease in cell viability and inhibition of colony formation. In contrast, healthy CD34^+^ cells appeared less sensitive to AC-4–130, indicating a therapeutic window. Remarkably, AC-4–130 showed promising in vivo activity, decreasing tumor growth in a subcutaneous AML xenograft model. This is of significant clinical importance, as up to 30% of AML patients harbor activating mutations in the receptor TK FLT3. Since the clinical impact of FLT3 inhibitors is limited due to transient responses and acquired resistance [34], the STAT5 inhibitor AC-4–130 represents a solid basis for further lead structure development towards compounds with clinical value for FLT3-ITD^+^ AML.

While TK-targeted therapy has been extremely successful in the treatment of hematopoietic neoplasia, the rapid emergence of resistance remains a challenge. One of the most important common pathways mediating resistance is JAK2/STAT5 signaling pathway activation, promoting cancer stem cell survival and self-renewal. Indeed, increased activation of STAT5 has been associated with TKI resistance [8, 35]. JAK2/STAT5 signaling is significantly increased in leukemic stem cells of high-risk AML patients [36]. Hence, targeted inhibition of different vulnerable nodes within the same core cancer pathway has great potential to eliminate cancer cells without causing resistance. In line, we found that the combination of AC-4–130 with the JAK1/2 inhibitor Ruxolitinib or the JAK2 inhibitor AG490 resulted in increased cytotoxicity in leukemic cells. We speculate that TKI treatment results in higher levels of uSTAT5, which in turn would increase the susceptibility of STAT5 to AC-4–130 inhibition.

STAT5 was shown to interact with chromatin remodeling proteins including TET1/2 [37], EZH2 [38], and p300/CBP [39]. The cellular phenotype induced by AC-4–130 might be the consequence of the disruption of multiple activities of STAT5, including various protein-protein interactions. We identified strong synergy between AC-4–130 and the p300/pCAF inhibitor Garcinol, as well as the CBP/p300 inhibitor I-CBP112. It is known that STAT5 acetylation by p300, and to a lesser extent by pCAF, plays an important role in regulating STAT5 phosphorylation and dimerization [40]. Thus, we speculate that the synergism between the two compounds arises from targeting STAT5 transcription from two angles. These observations suggest that small molecule STAT5 inhibitors not only represent a novel therapy, but they might also help to unravel undefined functions of STAT5 in AML cells.

In summary, we present AC-4–130 as a scaffold for further development of clinically relevant STAT5 inhibitors. Although direct targeting of transcription factors is challenging, AC-4–130 provides an important step forward to selectively target STAT5.

## Electronic supplementary material

Supplementary Material

Supplementary Figure 1

Supplementary Figure 2

Supplementary Figure 3

Supplementary Figure 4

Supplementary Figure 5

Supplementary Figure 6

Supplementary Figure 7

Supplementary Figure 8

Supplementary Figure 9

Supplementary Figure 10

Supplementary Figure 11
