# Supplementary material for: Pharmacologic inhibition of STAT5 in acute myeloid leukemia
Source: Leukemia. 2018 Feb 2;32(5):1135–46. doi: 10.1038/s41375-017-0005-9 (PMC5940656; doi:10.1038/s41375-017-0005-9)
Supplement: Supplementary file 1 — Supplementary Material [file 41375_2017_5_MOESM1_ESM.docx]

**Supplementary Materials and Methods**

**Amino acid sequence alignment, homology modeling & ligand docking**

Sequence alignments. Sequence alignments and visualization were done using Clustal Omega^1, 2^. Conserved amino acids are marked with black boxes.

Homology modeling. Mouse STAT5A (PDB ID: 1Y1U)^3^ was used as a structural template for the human STAT5B model. After preparing the template with the Protein Preparation Wizard^4^, the knowledge-based method of Prime was used to create the STAT5B model^5, 6^. The resulting models were evaluated by the corresponding Ramachandran plots^7^.

Ligand docking. The structure of the small-molecule ligand AC-4-130 in complex with wild-type STAT5B was modeled via ligand docking with Glide^8, 9^.

**^19^F NMR binding experiments**

^19^F NMR spectra were collected using a 600 MHz Varian Inova spectrometer equipped with an H(F)CN triple resonance cyroprobe. The buffer composition of all samples included 100 mM HEPES (pH 7.4), 2% glycerol, 10% D_2_O, 5% DMSO. For direct comparison, all samples were referenced to an added standard (100 μM 5-fluorotryptophan). STAT proteins were incubated with AC-4-130 for 2 h at 37°C and the 1D 19F spectra was recorded at 37°C.

**Thermal Shift Assays**

Thermal shift assays were performed as described previously^10^. Briefly, thermal shift assays were set up in triplicate using a C1000 Touch Thermocycler equipped with a CFX96 Real-time optical unit (Bio-rad, Hercules, CA, USA). In each assay, 200 nM protein and 2 μM of fluorescently labelled peptide (FAM-GpYLVLDKW, corresponding to the EPO receptor) were incubated for 10 min at room temperature in 100 mM HEPES (pH 7.4), 0.01% DMSO (v/v). Subsequently, 100 μM AC-4-130 or DMSO (control) was added to each well and allowed to incubate with the STAT5B:peptide complex for an additional 10 min. The samples were incrementally heated from 20 to 80°C in steps of 0.5°C with an equilibration time of 30 s in between each temperature. The fluorescence intensity was recorded at each temperature at 520 nm (slit width=10 nm) after excitation at 470 nm (slit width=20 nm). A negative first derivative plot was obtained from the emission intensity as a function of temperature, and the area under the curve was determined using a Riemann approximation.

**Cell culture & reagents**

All cell lines were maintained under standard conditions (95% humidity, 5% CO_2_, 37°C). Parental Ba/F3 and Ba/F3 FLT3-ITD cells were a kind gift from Dr. Böhmer (Jena, Germany). The human MV-4-11, MOLM-13 and HEK293T cells were purchased from DSMZ (Braunschweig, Germany). ML-2, PL-21, THP-1, A549, and HT-29 cells were provided by Dr. Grebien and Dr. Casanova (LBICR, Vienna, Austria).

Murine Ba/F3 cell lines and human cell lines were cultured in RPMI1640 supplemented with 10% FBS, 10 U/mL Penicillin, 10 µg/mL Streptomycin, 2 mM L-glutamine (all Gibco, Life Technologies, Carlsbad, CA, USA) and 2 µg/ml Ciprofloxacin (Sigma Aldrich, St. Louis, MO, USA). Parental Ba/F3 cells were maintained in the presence of 1 ng/ml mouse IL-3 (ImmunoTools, Friesoythe, Germany). Stat5^-/-^ MEFs were prepared from day E13.5-14.5 embryos from STAT5ab^+/-^ mice^11^ by mincing the carcass after removal of head and internal organs, digesting in 0.05% trypsin/0.53 mM EDTA (Gibco) for 10 min at 37°C, resuspending in complete growth medium (high glucose DMEM with 10% FCS, 2 mM L-Glutamine, 100 U/ml Penicillin-Streptomycin, 10 µg/ml Gentamicin, 0.1 M MEM Non-Essential Amino Acids, 55 µM β-Mercaptoethanol (all Gibco) and grown until confluent. HEK293T, A549, and HT-29 cells were maintained in DMEM (Gibco) supplemented with 10% FBS, 10 U/ml Penicillin, 10 µg/mL Streptomycin, 2 mM L-glutamine, and 2 µg/ml Ciprofloxacin. Human CD34^+^ cells were harvested from umbilical cord blood of healthy male and female donors using the EasySep™ Human CD34 Positive Selection Kit (Stemcell Technologies, Vancouver, BC, Canada). CD34^+^ enriched fractions were cultured in IMDM (Gibco) supplemented with 2.5% BSA (low endotoxin, PAN-Biotech, Aidenbach, Germany), 1x ITS-G (Gibco), 20 µM β-Mercaptoethanol (Acros Organics, Thermo Fisher Scientific, Waltham, MA, USA), 1 mM UltraGlutamine-I (Lonza, Basel, Switzerland), 1% Amphotericin B (250 µg/mL; PAN-Biotech), 1% Penicillin-Streptomycin (100x; Gibco), 50 µg/ml Gentamicin (Acros Organics), 10 µg/ml Ciprofloxacin (Acros Organics), 50 µM 2P-Ascorbic Acid (Sigma Aldrich), 1 µg/mL Heparin (AppliChem, Darmstadt, Germany), 0.5x Synthechol (500x; Sigma Aldrich), 50 ng/ml SCF (Peprotech, Rocky Hill, NJ, USA), 50 ng/ml FLT3L (Peprotech), 25 ng/ml TPO (Peprotech), and 35 nM UM171 (Selleckchem, Munich, Germany). Mycoplasma contamination was tested and excluded using the Venor GeM Classic Mycoplasma Detection Kit (Minerva Biolabs, Berlin, Germany). Bortezomib (Selleckchem), Garcinol (Cayman Chemical, Ann Arbor, MI, USA), Ruxolitinib (Eubio, Vienna, Austria), AG490 (Cayman Chemical), and I-CBP112 (Cayman Chemical) were dissolved in DMSO (Sigma) and diluted further in culture medium immediately before use.

**Primary AML patient samples**

Peripheral blood (PB) and/or BM cells (iliac crest or sternum) of patients with AML were collected at diagnosis and in follow-up. The study was approved by the ethics committee of the Medical University of Vienna and conducted in accordance with the declaration of Helsinki. All patients gave written informed consent. Diagnoses were established according to French-American-British (FAB) and World Health Organization (WHO) criteria^12^. Cells were maintained in RPMI1640 supplemented with 10% FBS, 10 U/mL Penicillin, 10 µg/mL Streptomycin, 2 mM L-glutamine and 2 µg/ml Ciprofloxacin. Patient material was examined for expression of CD34^+^/CD38^-^ AML LSCs via flow cytometry.

**Cytotoxicity assay**

1x10^4^ cells were plated in 96 well flat bottom plates and treated in triplicates with DMSO (Ctrl), Bortezomib (10 µM; positive control), or test compounds. Cell viability was assessed using the CellTiter Blue or CellTiter Glo assay (Promega, Madison, WI, USA) according to the manufacturer's protocol. IC_50_ values were determined using GraphPad Prism 5 by non-linear regression.

**Clonogenic assays in methylcellulose**

MV4-11 or MOLM-13 cells were plated in methylcellulose without additional cytokines (MethoCult^TM^ H4230, Stemcell Technologies) at a concentration of 1x10^3^ cells per plate and treated with AC-4-130 or DMSO (Ctrl). Patient samples were plated in methylcellulose supplemented with human cytokines and EPO (MethoCult H4435, Stemcell Technologies) at a concentration of 1x10^5^ cells per plate. All plates were incubated at 37°C, 5% CO2 for 10-14 days before counting the number of colonies.

**Apoptosis and cell cycle measurements**

The ratios of apoptotic cells were determined with the Annexin V-APC apoptosis detection kit (eBioscience, Vienna, Austria) according to the manufacturer’s protocol and analyzed by flow cytometry. Cell-cycle profiles were obtained by staining cells with propidium iodide (PI; 50 μg/ml) in hypotonic lysis solution (0.1% (w/v) sodium citrate, 0.1% (v/v) Triton X-100, 100 μg/ml RNAse) and incubating at 37°C for 30 min before analysis by flow cytometry.

**Caspase 3/7 activation assay**

MOLM-13 and MV4-11 cells were treated with AC-4-130 at indicated concentrations or DMSO (vehicle) for 24 h. Caspase 3/7 activity was captured using the Caspase-Glo® 3/7 Assay (Promega) according to the manufacturer’s protocol.

**Luciferase reporter gene assay**

5x10^6^ Ba/F3 cells were electroporated with pGL4.52[luc2P/STAT5 RE/Hygro] (*Firefly*, luciferase, Promega) and pRL-TK (*Renilla* luciferase). After 24 h, cells were starved overnight and pretreated with AC-4-130 or DMSO (Ctrl) for 6 h. Cells were stimulated with IL-3 (10 ng/ml) for 6 h, followed by measuring *Firefly* and *Renilla* luciferase activities using the dual-luciferase reporter assay system (Promega) following the manufacturer’s protocol. 5x10^6^ HT-29 cells were transfected with pGL4.47[luc2P/SIE/Hygro] and pRL-TK (*Renilla* luciferase) using Lipofectamine2000 (Thermo Scientific). After 24 h, cells were pretreated with AC-4-130 or DMSO (Ctrl) for 6 h. Cells were stimulated with IL-6 (10 ng/ml) or IFN-γ (10) for 16 h, followed by measuring *Firefly* and *Renilla* luciferase activities using the dual-luciferase reporter assay system (Promega) following the manufacturer’s protocol.

**Immunoblotting**

Sample preparation and Western blotting was performed using standard techniques. Nitrocellulose membranes (0.45 µm Amersham Protran 10600002, GE Healthcare, Buckinghamshire, UK) were incubated with the following antibodies in the dilution as indicated: Anti-phospho-STAT5 (Y694) polyclonal rabbit (71-6900; 1:1000-5000; Invitrogen, Camarillo, CA, USA), anti-STAT5 monoclonal mouse (sc-835; 1:5000; Santa Cruz, Dallas, TX, USA), anti-phospho-STAT3 (Y705) polyclonal rabbit (9131, 1:1000; CST; Danvers, MA, USA), anti-STAT3 monoclonal mouse (610189; 1:1000; BD Biosciences; Franklin Lakes, NJ, USA), anti-phospho STAT1 (Y701) polyclonal rabbit (9167; 1:1000; CST), anti-STAT1 monoclonal mouse (610185; 1:1000; BD Biosciences), anti- Cleaved Caspase-3 (Asp175) polyclonal rabbit (9661; 1:1000; CST), anti-PARP monoclonal rabbit (9532; 1:1000; CST), anti-HSC70 monoclonal mouse (sc-7298; 1:1000; Santa Cruz), anti-ß-ACTIN monoclonal mouse (A 5316; 1:10 000; Sigma Aldrich), ECL anti-rabbit HRP (NA934V) or anti-mouse (NA931) HRP (1:10.000; GE Healthcare).

**Determination of STAT5 dimerization by co-immunoprecipitation**

HEK293T cells (2x10^6^) were co-transfected with PCR cloned pMSCV STAT5A-FLAG and pMSCV STAT5A-MYC, verified for correct sequence by SANGER sequencing, using calcium phosphate precipitation according to the user manual (Calcium Phosphate Transfection Kit, Sigma Aldrich). After 24 h, cells were serum-starved for 6 h, pretreated with AC-4-130 at indicated concentrations or DMSO (vehicle) for 6 h, and stimulated with 500 ng/ml hGH (Immunotools) for 20 min. Cells were lysed in HE buffer (10 mM HEPES (pH 7.35), 1 mM EDTA) supplemented with protease inhibitors using a dounce tissue grinder. Dynabeads (Invitrogen) were washed in HE buffer. Whole cell lysates were immunoprecipitated with anti-MYC monoclonal mouse (sc-40; Santa Cruz) and immunoblotted with anti-FLAG® M2 monoclonal mouse (F3165; 1:1000, Sigma Aldrich), anti-MYC monoclonal mouse (1:1000, Santa Cruz), anti-STAT5A monoclonal mouse (sc-271542; 1:1000; Santa Cruz), and anti-HSC70 monoclonal mouse (sc-7298; 1:1000; Santa Cruz).

**Subcellular fractionation**

4x10^6^ Ba/F3 cells overexpressing FLT3-ITD were seeded and treated with AC-4-130 at different concentrations. Cells were incubated for 15 h and washed with PBS. Subsequently, drug treatment and washing was repeated twice and cells were incubated for 6 h each. To separate the nucleus from the cytoplasmic fraction, cells were suspended in buffer (10 mM HEPES (pH 7.9), 10 mM KCl, 0.1 mM EDTA, 0.1 mM EGTA, 2 mM DTT, 0.4 mM sodium orthovanadate, 25 mM sodium fluoride, 1 mM PMSF) and incubated on ice for 10 min. NP-40 was added to a final concentration of 0.6% to cells and cytoplasmic supernatant was collected by centrifugation (13000 g) for 60 sec.. The nuclear pellets were washed with PBS and suspended in buffer (20 mM HEPES (pH 7.9), 25% glycerol, 400 mM NaCl, 1 mM EDTA, 1 mM EGTA, 2 mM DTT, 0.4 mM sodium orthovanadate, 25 mM sodium fluoride, 1 mM PMSF) and vigorously shaken at 4°C for 30 min. Nuclear fraction was collected by centrifugation (13000 g) for 5 min. Proteins were separated on a SDS-PAGE gel and Western blotting was performed with the following antibodies: anti-LaminB1 (ab133741; Abcam, Cambridge, UK), anti-α-Tubulin (sc-32293; Santa Cruz), anti-STAT5 (sc-835; Santa Cruz), and anti-phospho STAT5 (611964; BD Transduction Laboratories, Franklin Lakes, NJ USA).

**RNA-seq processing, analysis and GSEA**

MV4-11 and MOLM-13 cells were treated in triplicates with 5 µM AC-4-130 or DMSO (Ctrl) for 24 h followed by total RNA extraction using the RNeasy Mini Kit (Qiagen, Venlo, Netherlands). RNA-seq 50 bp single-end libraries were sequenced on a HiSeq 2000 (Illumina, San Diego, CA, USA) machine, resulting in average 31.4 M reads per replicate. Alignments to genome version GRCh38.p10 and the corresponding Ensgene annotation were accomplished with STAR (STAR_2.5.0b)^13^ using default parameters. Expression level estimation was performed with featureCounts (version 1.5.0-p1)^14^ with the ‘-t exon’ option. Differential expression analysis was performed with DESeq2 (1.16.1)^15^. The design consisted of control and treatment triplicates of each cell line individually. Genes with low expression were filtered out if the size factor normalized row count was ≤1. The functions ‘DESeq()’ and ‘results()’ were then applied with default parameters. Venn Diagrams are based on significantly regulated genes with a P-value <0.01. Heatmaps were generated with centered and scaled rlog transformed counts using the heatmap.2 function of R package gplots (version 3.0.1)^16^. Differentially expressed genes (P-value <0.01) were further subsetted by intersection with the STAT5 hallmark gene set from the molecular signatures database (MSigDB)^17, 18^. Gene lists from differential expression analysis were ranked by log2 fold changes between untreated and treated cells. The data have been deposited to the GEO with the accession number GSE103510.

**mRNA isolation and quantitative real-time PCR**

MV4-11 and MOLM-13 cells were treated with 5 μM of AC-4-130 or DMSO (vehicle) for 24 h. RNA was isolated using TriZol (Invitrogen) and reverse transcription was performed using the RevertAid H Minus First Strand cDNA Synthesis Kit (Thermo Scientific) according to the manufacturer’s instructions. Quantitative real-time PCR was carried out in duplicates using SYBR Green on an Eppendorf Master-cycler RealPlex (Eppendorf, Hamburg, Germany). Results were normalized to *GAPDH* expression and quantified using the ΔC(T) method^19^. The sequences of the oligo-primers used are as follows:

*MYC* 5’-CACCGAGTCGTAGTCGAGGT-3’ and 5’-TTTCGGGTAGTGGAAAACCA-3’;

*BCL-2* 5’-GAGAAATCAAACAGAGGCCG-3’ and 5’-CTGAGTACCTGAACCGGCA-3’;

*CCND2* 5’-CTGCTGCATTGTTCCCATAG-3’ and 5’-TTCAGTGACCTGACATCCCA-3’;

*GAPDH* 5’-AATGAAGGGGTCATTGATGG-3’ and 5’-TTCAGTGACCTGACATCCCA-3’.

**Mouse xenograft studies and histopathology analysis**

Mice were maintained under pathogen-free conditions at the University of Veterinary Medicine, Vienna. Rag2^-/-^γc^-/-^ mice were on BALB/c background. Mice were at the age of 8-14 weeks at the time of cell implantation. All animal experiments were carried out according to the animal license protocol (BMWFW-68.205/0130-WF/V/3b/2016) approved by the Austrian Ministry BMWF authorities. The experimental design and number of mice assigned to each treatment arm were based on prior experience with similar models and provided sufficient statistical power to discern significant differences. Mice were matched according to initial tumor size and randomized to treatment with AC-4-130 or vehicle. No mice were excluded from the analysis. Blinding of experiments was not feasible in the experiments performed in this study.

*Safety assessment.* C57BL/6J mice were treated with AC-4-130 (25 mg/kg) or vehicle (10% DMSO, 5% Cremophore in saline buffer) by intraperitoneal injection daily for 21 days.

*Subcutaneous xenograft.* Mice were implanted subcutaneously in both flanks with 1x10^6^ human MV4-11 cells in 100 µl PBS, using a 27G needle. The animals were randomly divided into two groups and treated daily with AC-4-130 (25 mg/kg) or vehicle (10% DMSO, 5% Cremophore in saline buffer) from day 3 after engraftment until terminal workup. Tumor growth was measured twice a week using Vernier calipers for the duration of the treatment. Tumor volumes were calculated as the product of length x width x height. After termination of the experiment, tumors were resected and used for analysis of tumor weight, immunohistochemistry and immunoblotting as described.

*Hematocytometry and flow cytometry.* Blood was obtained by heart puncture and collected in EDTA-tubes (Mini-Collect K3EDTA tubes). WBC count and hematocrit were measured using an animal blood counter (sciI Vet abc, Viernheim, Germany). For flow cytometry, erythrocytes were lysed using Gay's solution (10 mM KHCO_3_ and 75 mM NH_4_Cl, pH 7.4). Flow cytometry was used in order to determine cellular components of blood, lymphnodes, and bone marrow. Single cell suspensions were prepared by mincing organs through a 70 µm-cell strainer (BD Biosciences, Franklin Lakes, NJ, USA). All flow cytometry antibodies used in this study are listed in Suppl. Table 1. All analyses were performed on a BD FACS Canto II™ instrument and calculated with FACSDiva software (v8.0.1, BD Biosciences).

*Histopathology and IHC.* Tumors were fixed overnight in 4% phosphate buffered formaldehyde solution (Roti^®^ Histofix, Carl Roth, Karlsruhe, Germany), dehydrated, paraffin embedded and cut. 3-µm-FFPE consecutive mouse organ or tumor sections were stained with Hematoxylin (Merck, Darmstadt, Germany) and Eosin G (Carl Roth). For immunohistochemical stainings, heat-mediated antigen retrieval was performed in citrate buffer at pH 6.0 (S1699; Dako, Agilent, Santa Clara, CA, USA) and sections were stained with antibodies against Ki67 (1:1000, Leica Biosystems, Wetzlar, Germany) and PDGFRβ (1:80; CST) using standard protocols.

*Measurement of biochemistry parameters.* Serum was prepared by centrifugation of the whole blood for 20 min at 7000 rpm. Serum concentration of aspartate aminotransferase (AST), alanine aminotransferase (ALT), creatinine (CRE), and blood urea nitrogen (BUN) was measured using a chemistry analyzer (IDEXX VetTest 8008, IDEXX GmbH, Ludwigsburg, Germany).

**Supplementary Table 1**

Flow cytometry antibodies (all antibodies were obtained from eBioscience unless indicated otherwise).

| Antigen | Fluorochrome | Catalogue No. |
| --- | --- | --- |
| CD19 | PE, eFluor® 450 | 12-0193-82, 48-0193-82 |
| CD3e | PerCP Cy5.5 | 45-0031-82 |
| CD4 | FITC, PE | 11-0041-82, 12-0041-82 |
| CD8a | FITC, PE, PerCP Cy5.5, APC | 11-0081-82, 12-0081-82, 45-0081-82, 17-0081-82 |
| Ly6.G (Gr1) | APC, eFluor® 450 | 17-5931-82, 48-5931-82 |
| CD11b | FITC, eFluor® 450 | 11-0112-82, 48-0112-82 |

**Supplementary Data References**

1. Goujon M, McWilliam H, Li W, Valentin F, Squizzato S, Paern J*, et al.* A new bioinformatics analysis tools framework at EMBL-EBI. *Nucleic acids research* 2010 Jul; **38**(Web Server issue)**:** W695-699.

2. Sievers F, Wilm A, Dineen D, Gibson TJ, Karplus K, Li W*, et al.* Fast, scalable generation of high-quality protein multiple sequence alignments using Clustal Omega. *Mol Syst Biol* 2011 Oct 11; **7:** 539.

3. Neculai D, Neculai AM, Verrier S, Straub K, Klumpp K, Pfitzner E*, et al.* Structure of the unphosphorylated STAT5a dimer. *The Journal of biological chemistry* 2005 Dec 9; **280**(49)**:** 40782-40787.

4. Sastry GM, Adzhigirey M, Day T, Annabhimoju R, Sherman W. Protein and ligand preparation: parameters, protocols, and influence on virtual screening enrichments. *Journal of computer-aided molecular design* 2013 Mar; **27**(3)**:** 221-234.

5. Jacobson MP, Pincus DL, Rapp CS, Day TJ, Honig B, Shaw DE*, et al.* A hierarchical approach to all-atom protein loop prediction. *Proteins* 2004 May 01; **55**(2)**:** 351-367.

6. Jacobson MP, Friesner RA, Xiang Z, Honig B. On the role of the crystal environment in determining protein side-chain conformations. *Journal of molecular biology* 2002 Jul 12; **320**(3)**:** 597-608.

7. Ramachandran GN, Ramakrishnan C, Sasisekharan V. Stereochemistry of polypeptide chain configurations. *Journal of molecular biology* 1963 Jul; **7:** 95-99.

8. Halgren TA, Murphy RB, Friesner RA, Beard HS, Frye LL, Pollard WT*, et al.* Glide: a new approach for rapid, accurate docking and scoring. 2. Enrichment factors in database screening. *Journal of medicinal chemistry* 2004 Mar 25; **47**(7)**:** 1750-1759.

9. Friesner RA, Banks JL, Murphy RB, Halgren TA, Klicic JJ, Mainz DT*, et al.* Glide: a new approach for rapid, accurate docking and scoring. 1. Method and assessment of docking accuracy. *Journal of medicinal chemistry* 2004 Mar 25; **47**(7)**:** 1739-1749.

10. de Araujo ED, Manaswiyoungkul P, Israelian J, Park J, Yuen K, Farhangi S*, et al.* High-throughput thermofluor-based assays for inhibitor screening of STAT SH2 domains. *J Pharm Biomed Anal* 2017 Sep 05; **143:** 159-167.

11. Cui Y, Riedlinger G, Miyoshi K, Tang W, Li C, Deng CX*, et al.* Inactivation of Stat5 in mouse mammary epithelium during pregnancy reveals distinct functions in cell proliferation, survival, and differentiation. *Molecular and cellular biology* 2004 Sep; **24**(18)**:** 8037-8047.

12. Bennett JM, Catovsky D, Daniel MT, Flandrin G, Galton DA, Gralnick HR*, et al.* Proposals for the classification of the acute leukaemias. French-American-British (FAB) co-operative group. *British journal of haematology* 1976 Aug; **33**(4)**:** 451-458.

13. Dobin A, Davis CA, Schlesinger F, Drenkow J, Zaleski C, Jha S*, et al.* STAR: ultrafast universal RNA-seq aligner. *Bioinformatics (Oxford, England)* 2013 Jan 01; **29**(1)**:** 15-21.

14. Liao Y, Smyth GK, Shi W. featureCounts: an efficient general purpose program for assigning sequence reads to genomic features. *Bioinformatics (Oxford, England)* 2014 Apr 01; **30**(7)**:** 923-930.

15. Love MI, Huber W, Anders S. Moderated estimation of fold change and dispersion for RNA-seq data with DESeq2. *Genome Biol* 2014; **15**(12)**:** 550.

16. Warnes GR, Bolker B, Bonebakker L, Gentleman R, Huber W, Liaw A*, et al.* gplots: Various R programming tools for plotting data. *R package version* 2009; **2**(4)**:** 1.

17. Subramanian A, Tamayo P, Mootha VK, Mukherjee S, Ebert BL, Gillette MA*, et al.* Gene set enrichment analysis: a knowledge-based approach for interpreting genome-wide expression profiles. *Proc Natl Acad Sci U S A* 2005 Oct 25; **102**(43)**:** 15545-15550.

18. Liberzon A, Birger C, Thorvaldsdottir H, Ghandi M, Mesirov JP, Tamayo P. The Molecular Signatures Database (MSigDB) hallmark gene set collection. *Cell Syst* 2015 Dec 23; **1**(6)**:** 417-425.

19. Livak KJ, Schmittgen TD. Analysis of relative gene expression data using real-time quantitative PCR and the 2(-Delta Delta C(T)) Method. *Methods* 2001 Dec; **25**(4)**:** 402-408.

**Supplementary Figure Legends**

**Supplementary Figure 1: Inhibition of the STAT5 SH2 domain with small-molecule salicylic acid based STAT5 inhibitors.** (**a**) Alignment of human STAT SH2 domains using Clustal Omega. Identical residues are indicated with black boxes. Similar residues are shaded with gray boxes and homologies are depicted as pairwise amino acid percent identity matrix (%). Amino acids involved in binding of AC-4-130 are indicated with an asterix. (**b**) Modified thermal shift assays of STAT5B, in the absence or presence of 100 μM AC-4-130, or 100 μM non-fluorescent-labelled peptide (positive Ctrl). (**c**) ^19^F NMR of STAT3 protein with the PFBS containing compound SH-4-54.

**Supplementary Figure 2: AC-4-130 efficacy is dependent on STAT5 activity levels.** (**a**) Western blot of Ba/F3 cell lines to show STAT5 expression and activity levels (pY-STAT5). β-ACTIN served as loading control. Blots are representative of 2 independent experiments. Uncropped version of the Western blot is shown in **Supplementary Fig. 10**. (**b**) Viability assay of Ba/F3 cell lines were treated in triplicates with indicated concentrations of AC-4-130 or DMSO (Ctrl) for 24 h. Cell viability was measured using CellTiter Blue assay. IC_50_ values were determined using GraphPad Prism 5 software (GraphPad Software, Inc.). (**c**) A positive correlation (Pearson correlation: r^2^=0.7114, p-value=0.0726) was found between STAT5 activity levels (pY-STAT5) and IC_50_ values. (**d**) Western blot of Ba/F3 cells lines treated with AC-4-130 or DMSO (Ctrl) for 6 h showing pY-STAT5 and STAT5. β-ACTIN served as loading control. Blots are representative of 2 independent experiments. Uncropped version of the Western blot is shown in **Supplementary Fig. 10**. (**e**) Western blot of HT-29 cells treated with AC-4-130 for 6 h showing pYSTAT3, STAT3, pYSTAT1, and STAT1. β-ACTIN served as loading control. Blots are representative of 2 independent experiments. Uncropped version of the Western blot is shown in **Supplementary Fig. 10**. (**f**) Quantification of pYSTAT5 and STAT5 protein levels in nuclear fractions.

**Supplementary Figure 3: AC-4-130 induces cell cycle arrest and subsequent apoptosis.** (**a**) MV4-11 and MOLM-13 cells were treated with AC-4-130 or DMSO (Ctrl) in a dose-dependent manner for 72 h. Apoptotic cells were detected by Annexin-V/PI staining followed by flow cytometric analysis. (**b**) Cells were treated with AC-4-130 or DMSO (Ctrl) for 24 h and immunoblotted for cleaved PARP and cleaved Caspase 3. HSC70 was used as loading control. Uncropped version of the Western blot is shown in **Supplementary Fig. 11**. (**c**) Caspase 3 and 7 activity was analyzed after 72 h treatment with AC-4-130 or DMSO (Ctrl) using the Caspase-Glo 3/7 assay. (**d**) MV4-11 and MOLM13 cells were treated with AC-4-130 or DMSO (Ctrl) as indicated for 72 h. Cell cycle distribution was determined using PI Staining followed by flow cytometric analysis as shown by representative blots. (**e**) MV4-11 or MOLM-13 cells were embedded in methylcellulose in the presence of DMSO (Ctrl) or AC-4-130. Colonies were counted 10 days after seeding. Representative pictures are depicted (magnification: 4x). Colony size was measured using ImageJ.

**Supplementary Figure 4: AC-4-130 inhibits STAT5 target gene expression.** (**a**) Venn-diagram of significantly down- and up-regulated genes (p-value ≤ 0.01) in MOLM-13 and MV4-11 cells treated with AC-4-130 (5 µM) or DMSO (Ctrl) for 24 h**.** (**b**) GSEA of differentially expressed genes in MOLM-13 cells. (**c**) Heatmap of differentially expressed genes in MOLM-13 cells enriched in the IL-2 STAT5 hallmark pathway.

**Supplementary Figure 5: AC-4-130 impairs clonogenic growth of human leukemic cells.** (**a**) Average of viability assays of AML patient samples or healthy controls treated with AC-4-130 or DMSO (Ctrl) for 48 h. (**b**) IC_50_ values of viability assays presented as mean ± SEM. (**c**) PBMC or BM samples were analyzed for the amount of CD34^+^ cells and correlated to the IC_50_ values obtained from the viability assay (Pearson correlation: r=0.3894, p-value=0.0171). (**d**) Average number of colonies formed from human AML patient cells or healthy controls (**e**) Human AML patient samples were treated with AC-4-130 or DMSO (Ctrl) for 48 h. Apoptotic cells were detected by AnnexinV/PI staining. Representative dot plots are shown.

**Supplementary Figure 6: Healthy mice tolerate AC-4-130 treatment.** Healthy C57BL/6J mice were treated with AC-4-130 (25 mg/kg) or vehicle for 21 days by daily intraperitoneal injection. (**a**) Body weight (**b**) WBC count and hematocrit were measured. (**c**) Flow cytometric analysis of granulocyte, T- or B-cell numbers in blood, bone marrow or lymph nodes, as well as cancer stem cell/LSK cells in the bone marrow. (**d**) Plasma levels of ALT, AST, or BUN revealed normal levels. (**e**) Representative pictures of MV4-11 tumors (left) and tumor weight (right). (**f**) Quantification of pYSTAT5 levels immunoblots showing pY-STAT5 and STAT5 levels of xenograft tumors after treatment.

**Supplementary Figure 7: A drug screen reveals synergism with TK inhibitors and cytotoxic drugs.** (**a**) MV4-11 and MOLM-13 cells were treated in triplicates with single drugs or drug combinations as indicated for 24 h. Cell viability was analyzed using CellTiter Glo Assay.

**Supplementary Figure 8: Uncropped blot corresponding to main Figure 2.** Uncropped versions of Western blots from main Figure 2a and 2d are shown, as indicated.

**Supplementary Figure 9: Uncropped blot corresponding to main Figure 6.** Uncropped versions of Western blots from main Figure 6b are shown, as indicated.

**Supplementary Figure 10: Uncropped blot corresponding to Supplementary Figure 2.** Uncropped versions of Western blots from Supplementary Figure 2a, 2d and 2e are shown, as indicated.

**Supplementary Figure 11: Uncropped blot corresponding to Supplementary Figure 3.** Uncropped versions of Western blots from Supplementary Figure 3b are shown, as indicated.

**Supplementary Notes**

**Synthesis protocol**

Anhydrous solvents methanol, DMSO, CH_2_Cl_2_, THF and DMF were purchased from Sigma Aldrich and used directly from Sure-Seal bottles. Molecular sieves were activated by heating to 300°C under vacuum overnight. All reactions were performed under an atmosphere of dry nitrogen in oven-dried glassware and were monitored for completeness by thin-layer chromatography (TLC) using silica gel (visualized by UV light, or developed by treatment with KMnO4 stain). NMR spectra were recorded in Bruker Avance III spectrometer at 23°C, operating at 400 MHz for 1H NMR and 100 MHz 13C NMR spectroscopy either in CDCl_3_, CD_3_OD or d6-DMSO. Chemical shifts (δ) are reported in parts per million (ppm) after calibration to residual isotopic solvent. Coupling constants (J) are reported in Hz. High Resolution Mass Spectrometry (HRMS) was performed on an AB/Sciex QStar mass spectrometer with an ESI source, MS/MS and accurate mass capabilities, associated with an Agilent 1100 capillary LC system. Low Resolution Mass Spectrometry (LRMS) was performed on a Waters Micromass ZQ model MM1. Before biological testing, inhibitor purity was evaluated by reversed-phase HPLC (rpHPLC). Analysis by rpHPLC was performed using a Phenomenex Luna 5u C18 150 mm x 4.6 mm column run at 1.2 mL/min, and using gradient mixtures. The linear gradient consisted of a changing solvent composition of either (I) 15% MeCN and 85% H_2_O with 0.1% TFA (v/v) to 100% MeCN over 30 minutes and (II) 15% MeCN and 85% H_2_O with 0.1% TFA (v/v) to 100% MeCN over 60 minutes, UV detection at 250 nm. For reporting HPLC data, percentage purity is given in parentheses after the retention time for each condition. All biologically evaluated compounds are >95% chemical purity as measured by HPLC.


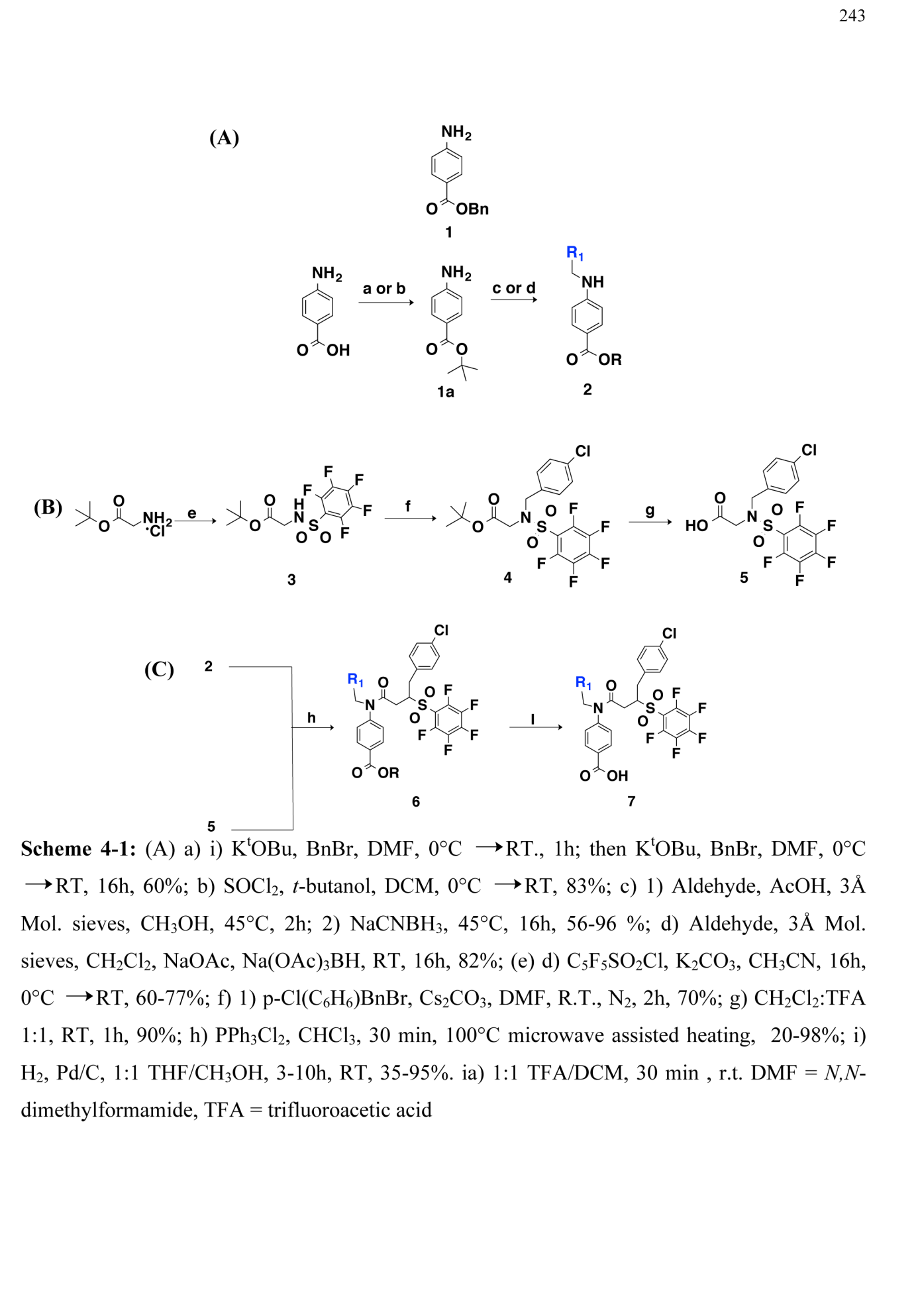


**Step 1:** *Procedure a:* KtOBu, BnBr, DMF, 0°C RT, 1h; then KtOBu, BnBr, DMF, 0°C RT, 16 h, 60%; *Procedure b:* SOCl_2_, t-butanol, DCM, 0°C RT, 83%; *Procedure c:* Aldehyde, AcOH, 3Å Mol. sieves, CH_3_OH, 45°C, 2 h; then NaCNBH_3_, 45°C, 16h, 56-96%; *Procedure d:* Aldehyde, 3Å Mol. sieves, CH_2_Cl_2_, NaOAc, Na(OAc)_3_BH, RT, 16 h, 82%;


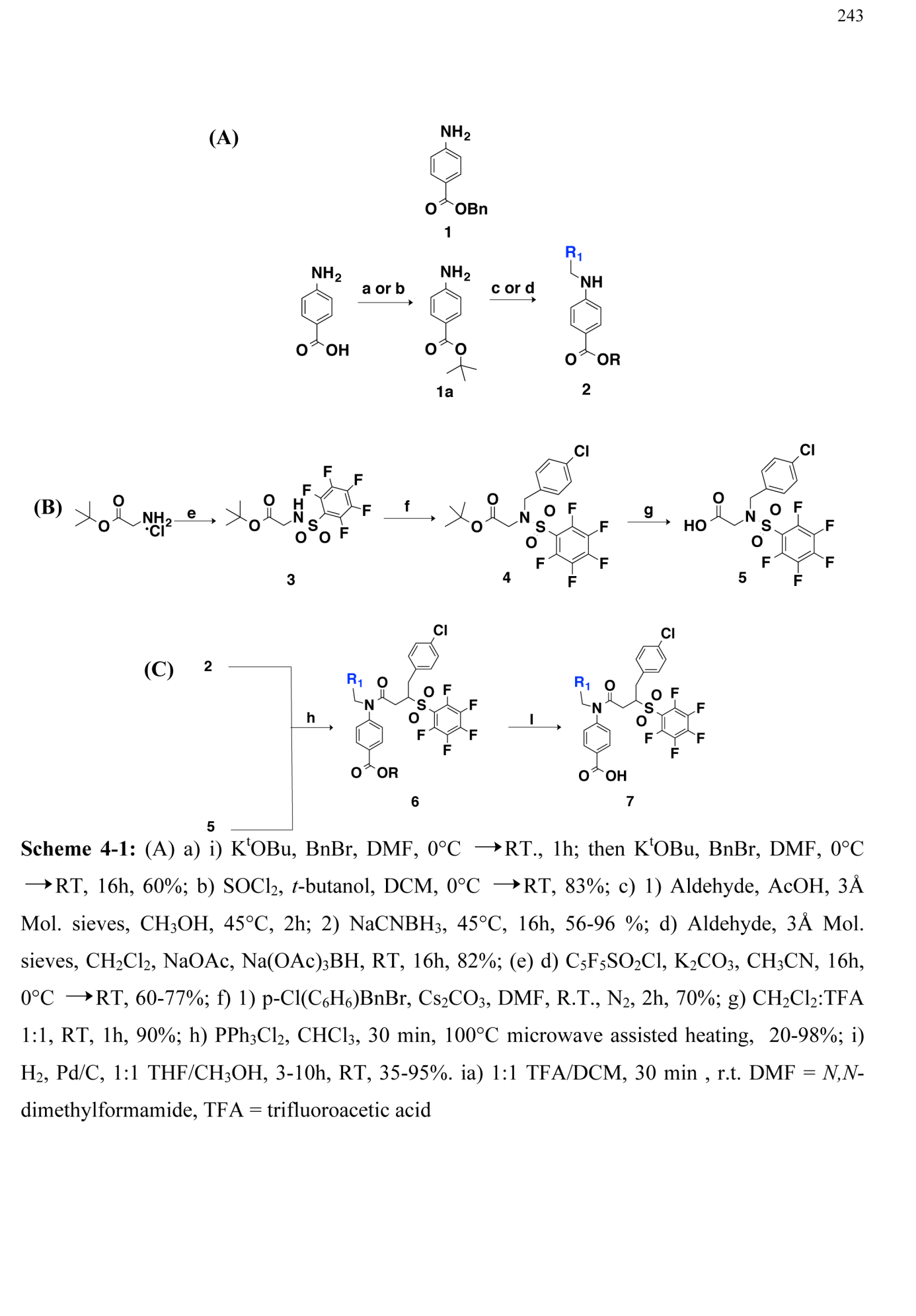


**Step 2:** *Procedure e:* C_5_F_5_SO_2_Cl, K_2_CO_3_, CH_3_CN, 16 h, 0°C RT, 60-77%; *Procedure f:* p-Cl(C_6_H_6_)BnBr, Cs_2_CO_3_, DMF, R.T., N_2_, 2 h, 70%; *Procedure g:* CH_2_Cl_2_:TFA 1:1, RT, 1 h, 90%;


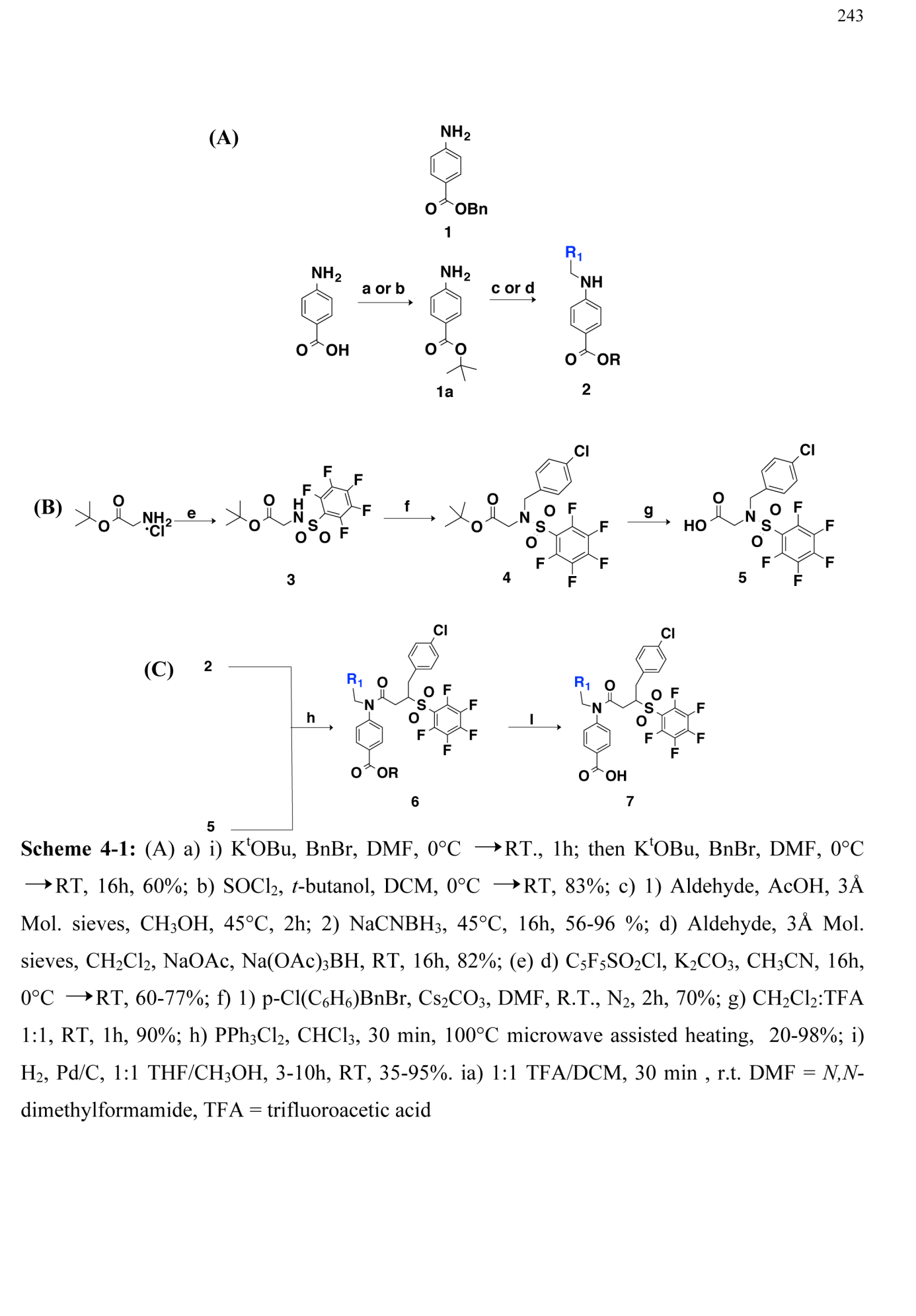


**Step 3:** *Procedure h:* PPh_3_Cl_2_, CHCl_3_, 30 min, 100°C microwave assisted heating, 20-98%; P*rocedure i:* H2, Pd/C, 1:1 THF/CH_3_OH, 3-10 h, RT, 35-95%; 1:1 TFA/DCM, 30 min, r.t. DMF=N,Ndimethylformamide, TFA=trifluoroacetic acid

**Procedure a: dibenzylation of salicylic acid.** To a stirred solution of 4-aminosalicylic acid (1.0 eq) in DMF (0.1 M) at 0°C was added KOtBu (1.2 eq). After 15 min, benzyl bromide (1.2 eq) was added drop-wise. The suspension was allowed to stir at room temperature for a further 4 h before the reaction vessel was again cooled to 0°C. Once again, KOtBu (1.2 eq) was added prior to the dropwise addition of benzyl bromide (1.2 eq). The reaction was left to stir overnight before quenching with H_2_O. The solution was then repeatedly extracted with ethyl acetate and the organic phases combined. The organics were then washed with H_2_O and brine then concentrated, dried over Na_2_SO_4_ and concentrated in vacuo. The resulting residue was purified using the Biotage Isolera automated column chromotographer with a 4:1 Hexanes/EtOAc gradient under reduced pressure.

**Procedure b: t-butyl protection of salicylic acid.** 4-aminobenzoic acid was suspended in SOCl_2_ (2.0 eq.) at 25°C. The suspension was refluxed for 2 h. SOCl_2_ was removed under reduced pressure, the last traces by azeotrope with CHCl_3_ (x3). The resulting acid chloride was dissolved in CH_2_Cl_2_ and a solution of t-butanol in CH_2_Cl_2_ was added to the stirred solution which was cooled to 0°C. A solid white precipitate was formed – the hydrochloride salt was quenched with 1.0 M KOH and extracted with EtOAC (x4).

**Procedure c: Reductive amination using sodium triacetoxyborohydride.** To a solution of protected 4-amino salicylic acid 1/1a (1.2 eq) and acetic acid (1.2 eq) stirred in anhydrous dichloroethane (0.1 M) with 3 Å mol. sieves was added the aldehyde (1.0 eq). The solution was then stirred at RT for 5 min after which Na(OAc)_3_BH (1.5 eq) was added and left to stir at RT overnight. The reaction was diluted with CH_2_Cl_2_, filtered and concentrated in vacuo. The concentrate was purified using the Biotage Isolera automated column chromatographer using a gradient of Hexanes/EtOAc to furnish secondary aniline derivatives 2.

**Procedure d: Sulfonylation of glycine and sarcosine t-butyl ester.** A solution of amino acid t-butyl ester (1 eq) and K_2_CO_3_ (1.1 eq) were dissolved in anhydrous acetonitrile and cooled to 0°C before sulfonyl chloride (1 eq) was added. The resultant solution was allowed to stir overnight at RT. The reaction was concentrated in vacuo and residue was dissolved in CH_2_Cl_2_. The organics were combined and then washed sequentially with 0.1 M HCl, saturated NaHCO_3_ and brine. The organics were then dried over Na_2_SO_4_ and concentrated in vacuo to furnish derivatives 3 with no further purification.

**Procedure e: Alkylation of sulfonamide.** A solution of 3 (1 eq) and Cs_2_CO_3_ (1.3 eq) were dissolved in anhydrous DMF followed by the addition of p-ClBnBr (1 eq). The resultant solution was allowed to stir for 2 h at RT. The reaction was quenched with H_2_O and then repeatedly extracted with ethyl acetate. The organic phases were combined and washed with H_2_O, brine, dried over Na_2_SO_4_ and concentrated in vacuo. The resulting residue was purified using the Biotage Isolera automated column chromotographer with a 2:1 Hexanes/EtOAc gradient under reduced pressure to provide **product** 4.

**Procedure f: t-butyl ester deprotection.** A solution of amino acid t-butyl ester (1 eq) was dissolved in TFA and immediately diluted with CH_2_Cl_2_ in a 1:1 mixture (0.1 M) solution. The resultant solution was allowed to stir for 1h and then co-evaporated with CH_3_OH (3x) and CHCl_3_ (3x). The resultant residue was carried forward with no purification to yield **product 5**.

**Procedure g: PPh3Cl_2_ peptide coupling.** To a stirred solution of carboxylic acid (5) (1.1 eq) in CHCl_3_ (0.1 M) was added PPh_3_Cl_2_ (2.5 eq). The reaction was allowed to stir 5 min at RT or until complete dissolvation, followed by the drop-wise addition of the secondary aniline 2 (1.0 eq). The reaction mixture was then heated in a microwave at 100°C for 30 min. The reaction mixture was allowed to cool to RT followed by sequential washing with saturated NaHCO_3_ and brine. The organic layers were then dried over Na_2_SO_4_ and concentrated in vacuo. The concentrate was absorbed directly onto silica for column chromatography purification using a gradient of hexanes and EtOAc to furnish **product 6**.

**Procedure h: Hydrogenolysis of the benzyl ester and benzyl ether.** The benzyl protected salicylic acid (**product 6**; 1 eq) was dissolved in a stirred solution of CH_3_OH/THF (1:2, 0.1 M). The solution was thoroughly degassed before the careful addition of 10% Pd/C (10 mg/mmol). H_2_ gas was bubbled through the solvent for 5 min before the solution was put under an atmosphere of H_2_ gas and stirred continuously for 2-5 h, monitoring completion of reaction via TLC. The H_2_ gas was evacuated and the reaction filtered through celite to remove Pd catalyst and concentrated in vacuo. The resulting residue was adsorbed onto silica and columned using a Biotage Isolera in a gradient of CH_2_Cl_2_, CH_3_OH and 1% acetic acid.

**Procedure i: Acid deprotection of t-butyl functional groups.** The t-butyl protected salicylic acid, was dissolved with 1:1 ration of TFA/DCM and stirred for 30 min. The crude solution was concentrated down in vacuo. The resulting residue was adsorbed onto silica and columned using a Biotage Isolera in a gradient of CH_2_Cl_2_, CH_3_OH and 1% acetic acid to provide final molecule **AC-4-130**.

**Intermediate characterization data**

**

**

**Benzyl 4-amino-2-(benzyloxy)benzoate (product 1a)** was synthesized according to **procedure a**, yielding the final product as an orange solid (47%): δH (400 MHz, d- CDCl3) 5.07 (s, 2H, CH2), 5.21 (s, 2H, CH_2_), 5.99 (br s, 2H, NH_2_), 6.18 (dd, J=8.6 and 1.8 Hz, 1H, CH)), 6.32 (d, J=1.7 Hz, 1H, CH), 7.28-7.38 (8H, m, CH), 7.47 (d, J=7.2 Hz, 2H, CH), 7.60 (d, J=8.6 Hz, 1H, CH);; δC (400 MHz, CDCl_3_) 65.8, 70.2, 99.1, 106.7, 109.0, 126.8, 127.5, 127.7, 127.9, 128.3, 128.4, 134.3, 136.6, 136.7, 152.2, 160.7, 165.7; LRMS (ESI+) calculated for C_21_H_19_NO_3_ [M + H]+ 333.2, found 333.2.

**

**

**Tert-butyl 4-aminobenzoate (product 1b)** was synthesized according to **procedure b** on (83%): 1H NMR (400 MHz, DMSO-d6) δ 1.06 – 1.19 (m, 7H), 1.39 (s, 1H), 1.46 (s, 16H), 1.52 (d, J=3.6 Hz, 3H), 1.88 (s, 1H), 1.96 (s, 6H), 4.00 (q, J=7.1 Hz, 4H), 5.82 (d, J=15.3 Hz, 4H), 6.47 – 6.55 (m, 4H), 7.50 – 7.59 (m, 4H), 7.79 – 7.98 (m, 2H). 13C NMR (101 MHz, DMSO-d6) δ 28.16, 28.30, 79.14, 112.80, 117.95, 119.36, 119.66, 129.89, 131.18, 153.37, 165.58. LRMS (ESI+) calculated for C_11_H_16_NO_2_ [M + H]+ 194.11, found 194.15.





**Benzyl 4-((3,5-di-tert-butylbenzyl)amino)benzoate (product 2a)** synthesized according to **procedure c** on (60%): 1H NMR (400 MHz, Chloroform-d) δ 1.26 (d, J=7.0 Hz, 6H), 2.91 (hept, J=6.9 Hz, 1H), 4.34 (s, 2H), 5.32 (s, 2H), 6.59 (dd, J=8.5, 1.2 Hz, 2H), 7.22 (d, J=7.9 Hz, 2H), 7.24 – 7.40 (m, 4H), 7.44 (d, J=7.9 Hz, 2H), 7.88 – 7.95 (m, 2H). 13C NMR (101 MHz, Chloroform-d) δ 23.87, 33.68, 47.31, 65.82, 111.48, 118.30, 126.69, 127.39, 127.81, 127.84, 128.35, 131.59, 135.47, 136.56, 148.18, 151.76. LRMS (ESI+) calculated for C_29_H_36_NO_2_ [M + H]+ 430.26, found 430.28.

**

**

**Tert-butyl 4-((3,5-di-tert-butylbenzyl)amino)benzoate (product 2b)** synthesized according to general **procedure c** on (85%): 1H NMR (400 MHz, Chloroform-d) δ 1.47 (s, 18H), 1.68 (s, 9H), 4.43 (d, J=5.1 Hz, 2H), 4.67 (t, J=5.3 Hz, 1H), 6.66 – 6.73 (m, 2H), 7.32 (d, J=1.8 Hz, 2H), 7.50 (t, J=1.8 Hz, 1H), 7.93 – 8.00 (m, 2H). 13C NMR (101 MHz, Chloroform-d) δ 28.34, 31.49, 34.82, 48.43, 79.64, 111.45, 120.27, 121.47, 121.90, 131.32, 137.58, 151.18, 151.74, 166.15. LRMS (ESI+) calculated for C_26_H_38_NO_2_ [M + H]+ 396.28, found 396.30.





**tert-butyl ((perfluorophenyl)sulfonyl)glycinate (product 3).** Derivative 3 synthesized using **procedure d** (71%): δH (400 MHz, CDCl_3_) 1.40 (s, 9H, COO(CH_3_)_3_), 3.93 (s, 2H, CH_2_), 5.54 (s, 1H, NH); δC (400 MHz, CDCl_3_) 27.5, 45.1, 82.8, 115.6, 136.5, 144.8, 145.9, 167.0; LRMS (ESI+) calculated for C_12_H_12_F_5_NO_4_S [M + H]+ 361.0, found 362.1.

**

**

**tert-butyl N-(4-chlorobenzyl)-N-((perfluorophenyl)sulfonyl)glycinate (product 4).** Derivative 4 synthesized using **procedure e** (71%): δH (400 MHz, CDCl_3_) 1.37 (s, 9H, (CH_3_)_3_), 3.93 (s, 2H, CH_2_), 4.57 (s, 2H, CH_2_), 7.24 (d, J=8.5 Hz, 2H, CH), 7.33 (d, J=8.5 Hz, 2H, CH); δC (400 MHz, CDCl_3_) 27.5, 52.5, 53.0, 81.8, 112.6, 126.5, 130.1, 132.4, 134.3, 136.5, 144.8, 145.9, 167.0; LRMS (ESI+) calculated for C_19_H_17_F_5_ClNO_4_S [M + H]+ 485.0, found 486.1.

**

**

**N-(4-chlorobenzyl)-N-((perfluorophenyl)sulfonyl)glycine (product 5).** Derivative 5 synthesized using **procedure f** (92%): δH (400 MHz, CDCl3) 4.13 (s, 2H, CH_2_), 4.58 (s, 2H, CH_2_), 7.25 (d, J=8.2 Hz, 2H, CH), 7.35 (d, J=8.2 Hz, 2H, CH); δC (400 MHz, CDCl_3_) 52.8, 54.2, 112.6, 126.5, 130.1, 132.4, 134.3, 136.5, 144.8, 145.9, 172.0; LRMS (ESI+) calculated for C_15_H_9_F_5_ClNO_4_S [M + H]+ 428.9, found 429.9.





**tert-butyl 4-(2-((N-(4-chlorobenzyl)-2,3,4,5,6-pentafluorophenyl)sulfonamido)-N-(3,5-di tertbutylbenzyl) acetamido)benzoate (product 6)** synthesized using **procedure g** (78%). 1H NMR (400 MHz, Chloroform-d) δ 1H NMR (400 MHz, Chloroform-d) δ 1.24 (s, 18H), 1.56 (s, 9H), 3.52 – 3.87 (m, 2H), 4.61 (s, 2H), 4.74 (d, J=12.3 Hz, 2H), 6.71 (d, J=8.0 Hz, 2H), 6.81 – 6.87 (m, 2H), 7.14 (d, J=8.5 Hz, 2H), 7.20 – 7.34 (m, 3H), 7.84 (d, J=8.0 Hz, 2H). 13C NMR (101 MHz, Chloroform-d) δ 27.94, 31.16, 34.55, 47.68, 50.26, 53.34, 81.60, 121.45, 123.10, 127.83, 128.91, 129.97, 130.81, 132.22, 132.62, 134.28, 134.55, 150.92, 164.26, 165.33. LRMS (ESI+) calculated for C_41_H_45_ClF_5_N_2_O_5_S [M + H]+ 807.26 found 807.29.





**4-(2-((N-(4-chlorobenzyl)-2,3,4,5,6-pentafluorophenyl)sulfonamido)-N-(3,5-di-tertbutylbenzyl) acetamido)benzoic acid (AC-4-130)** synthesized using **procedure h and i**: 1H NMR (400 MHz, Chloroform-d) δ 1.24 (d, J=1.1 Hz, 10H), 4.62 (s, 1H), 4.75 (s, 1H), 6.77 (d, J=8.0 Hz, 1H), 6.85 (s, 1H), 7.15 (d, J=8.1 Hz, 1H), 7.18 – 7.28 (m, 2H), 7.28 – 7.34 (m, 1H), 7.96 (d, J=8.0 Hz, 1H). 13C NMR (101 MHz, Chloroform-d) δ 31.32, 34.72, 47.87, 50.42, 53.54, 77.21, 121.73, 123.33, 128.41, 129.09, 130.15, 131.74, 132.69, 134.51, 146.24, 151.19, 165.39, 169.84. HRMS (ESI-) calculated for [C_37_H_35_ClF_5_N_2_O_5_S]-749.1881, found 749.1902.
