# Supplementary figures and images for: Pharmacologic inhibition of STAT5 in acute myeloid leukemia
Source: Leukemia. 2018 Feb 2;32(5):1135–46. doi: 10.1038/s41375-017-0005-9 (PMC5940656; doi:10.1038/s41375-017-0005-9)

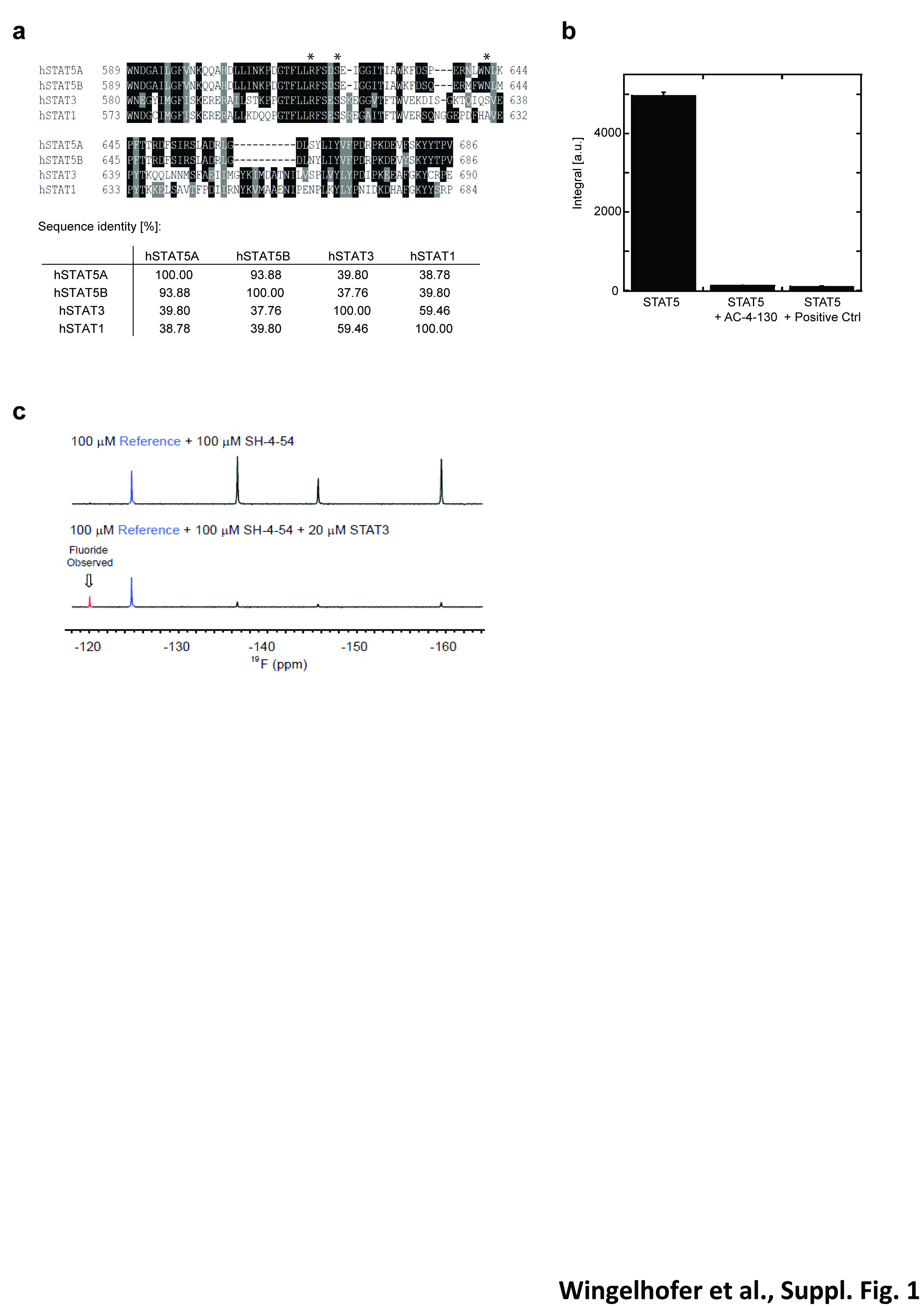

Supplement: Supplementary file 2 — Supplementary Figure 1 [file 41375_2017_5_MOESM2_ESM.tif]

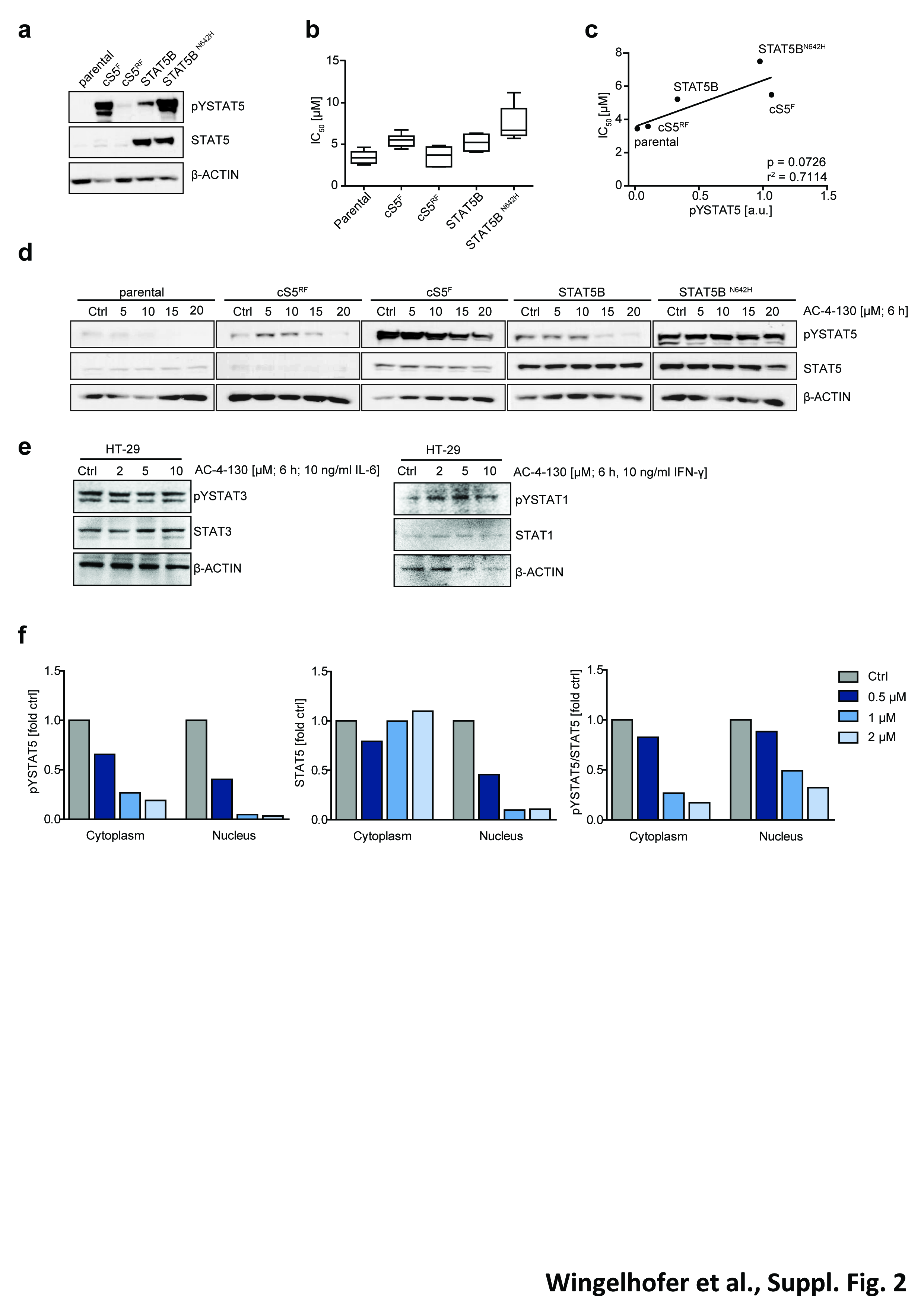

Supplement: Supplementary file 3 — Supplementary Figure 2 [file 41375_2017_5_MOESM3_ESM.tif]

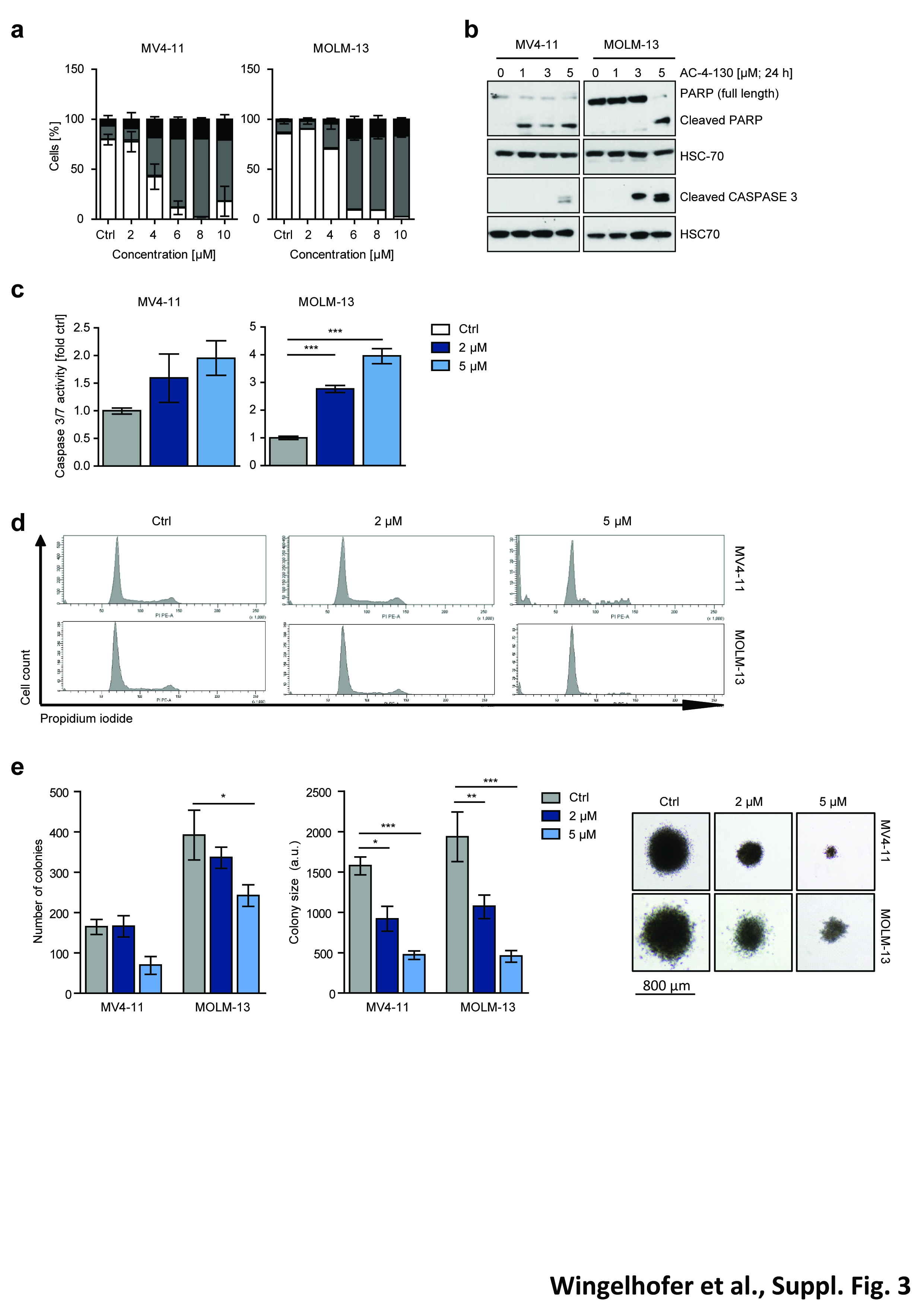

Supplement: Supplementary file 4 — Supplementary Figure 3 [file 41375_2017_5_MOESM4_ESM.tif]

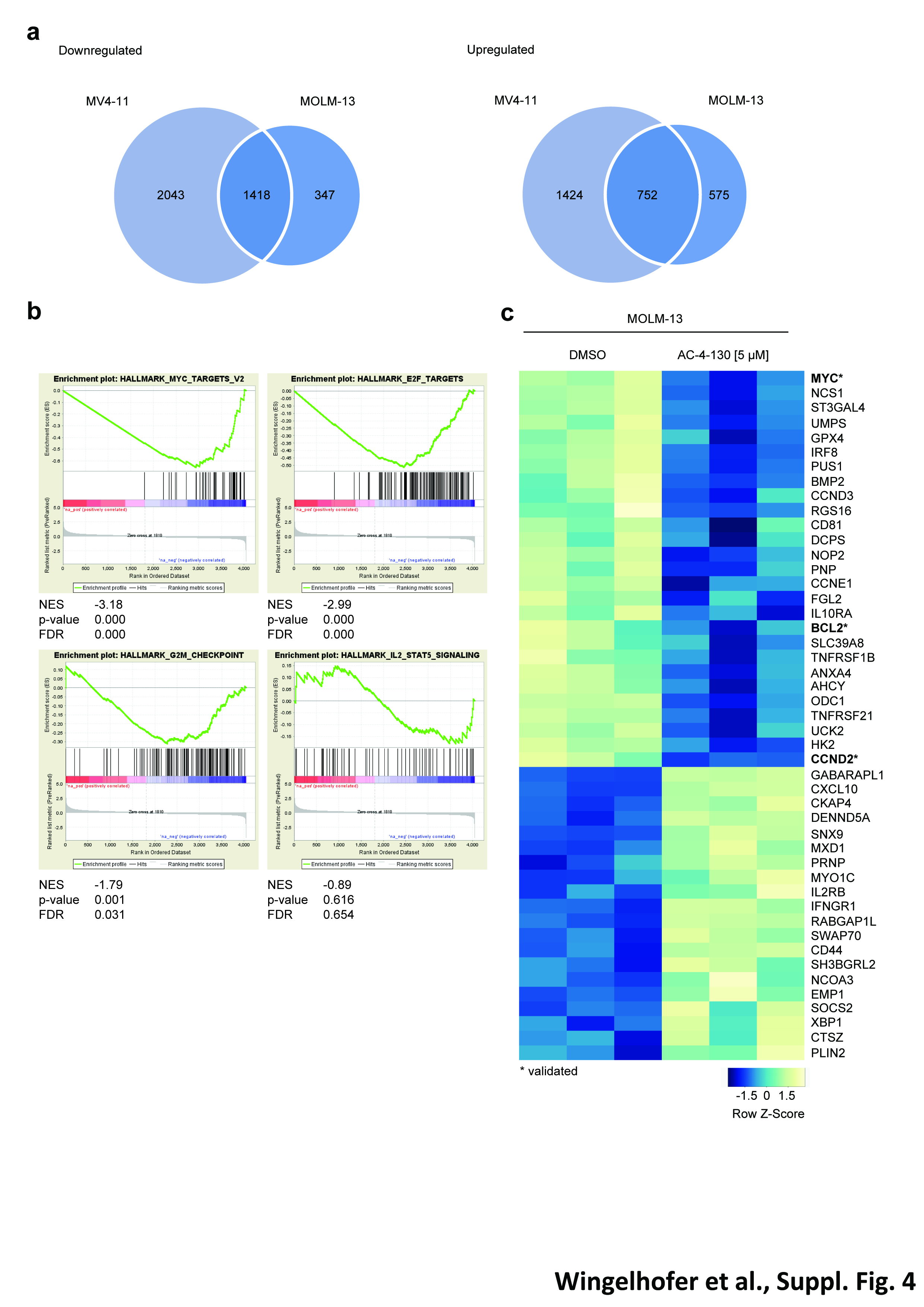

Supplement: Supplementary file 5 — Supplementary Figure 4 [file 41375_2017_5_MOESM5_ESM.tif]

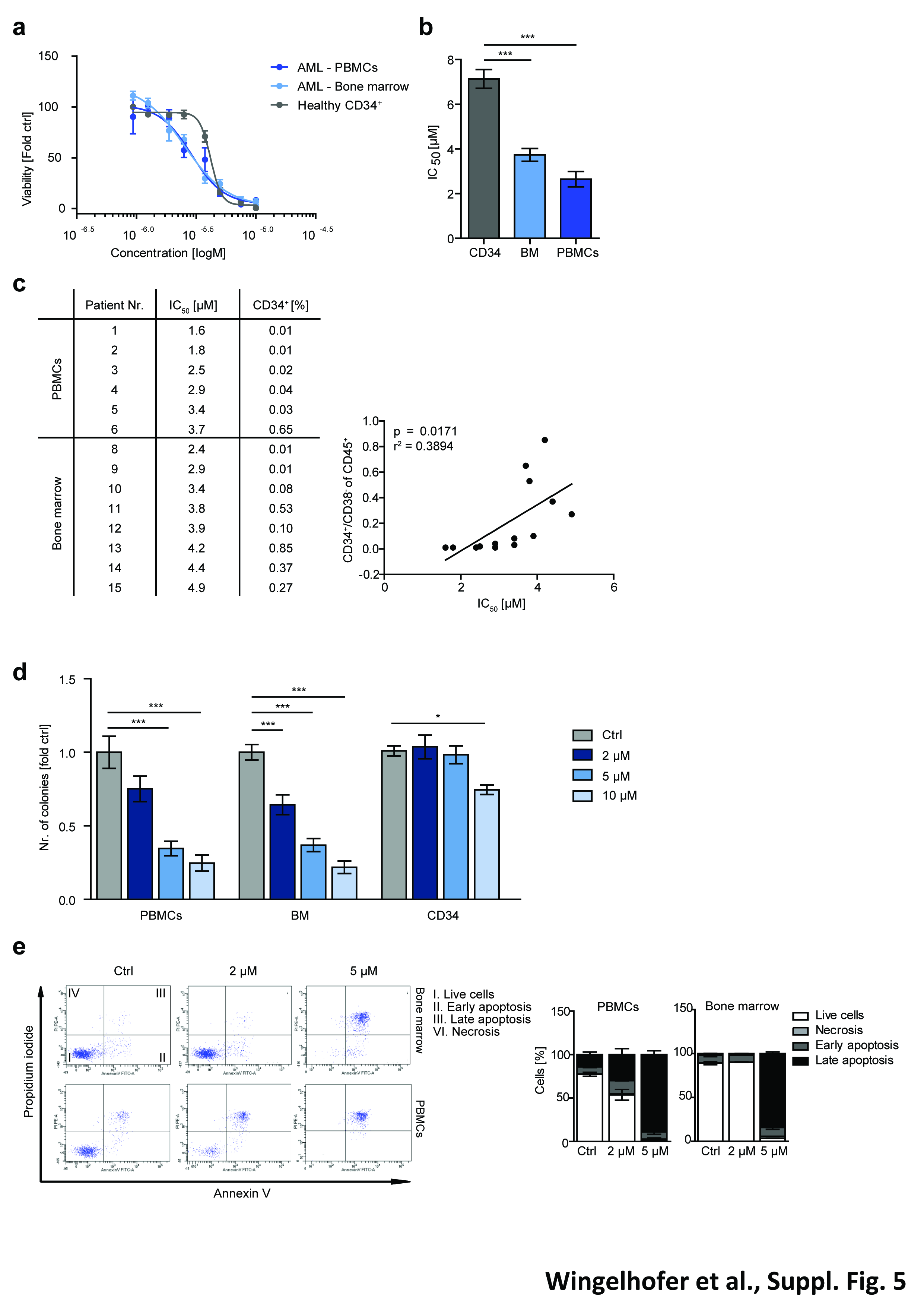

Supplement: Supplementary file 6 — Supplementary Figure 5 [file 41375_2017_5_MOESM6_ESM.tif]

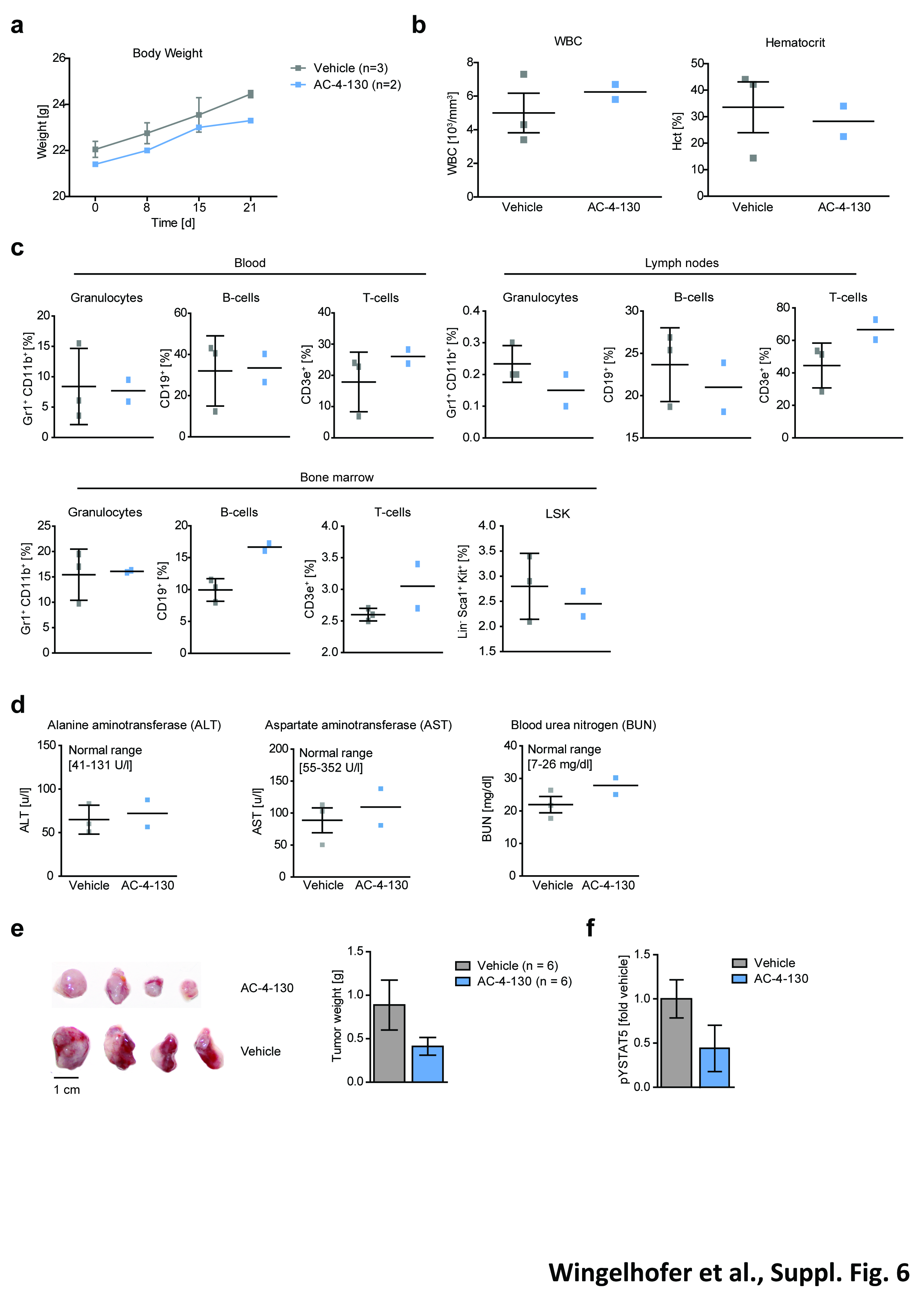

Supplement: Supplementary file 7 — Supplementary Figure 6 [file 41375_2017_5_MOESM7_ESM.tif]

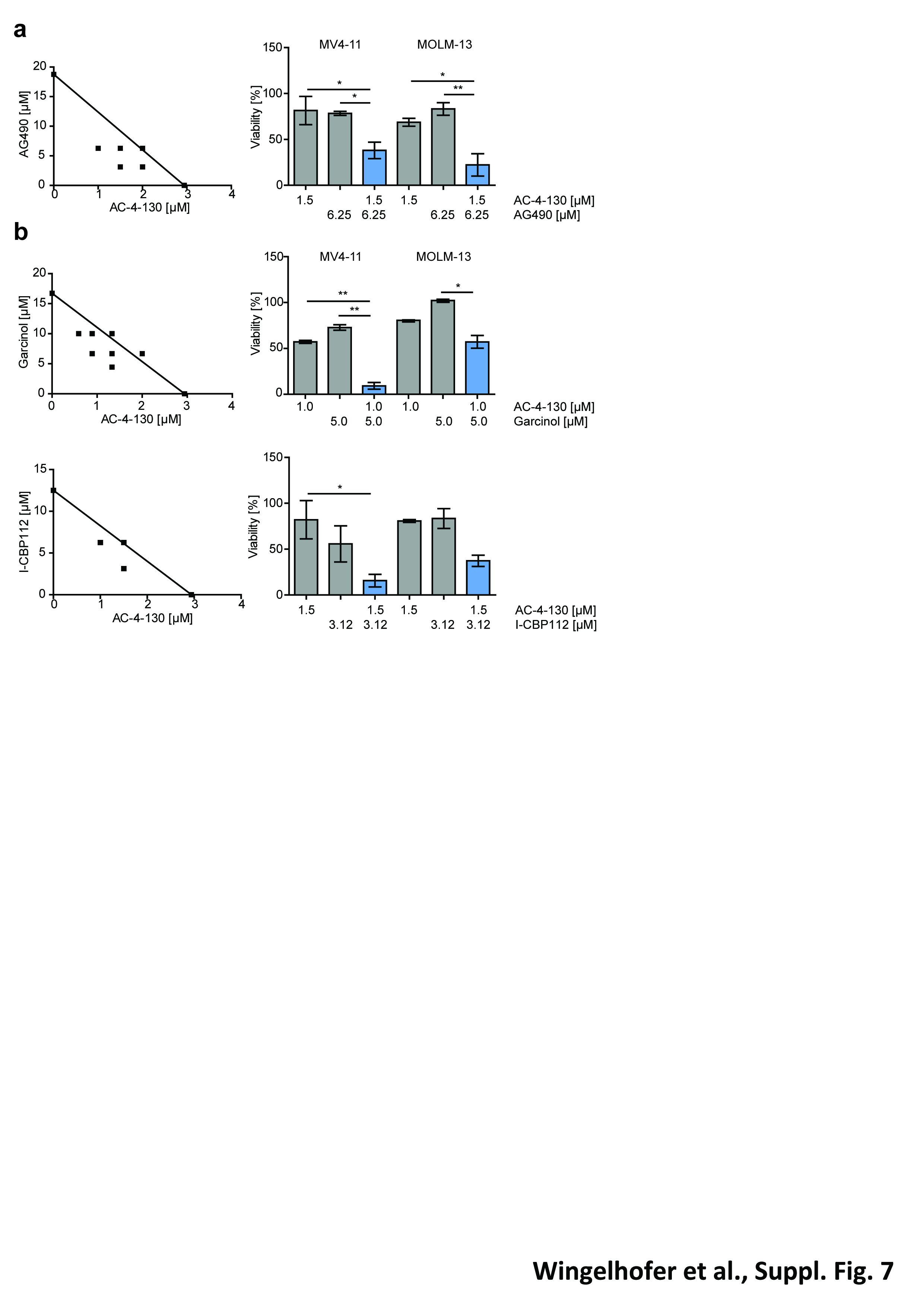

Supplement: Supplementary file 8 — Supplementary Figure 7 [file 41375_2017_5_MOESM8_ESM.tif]

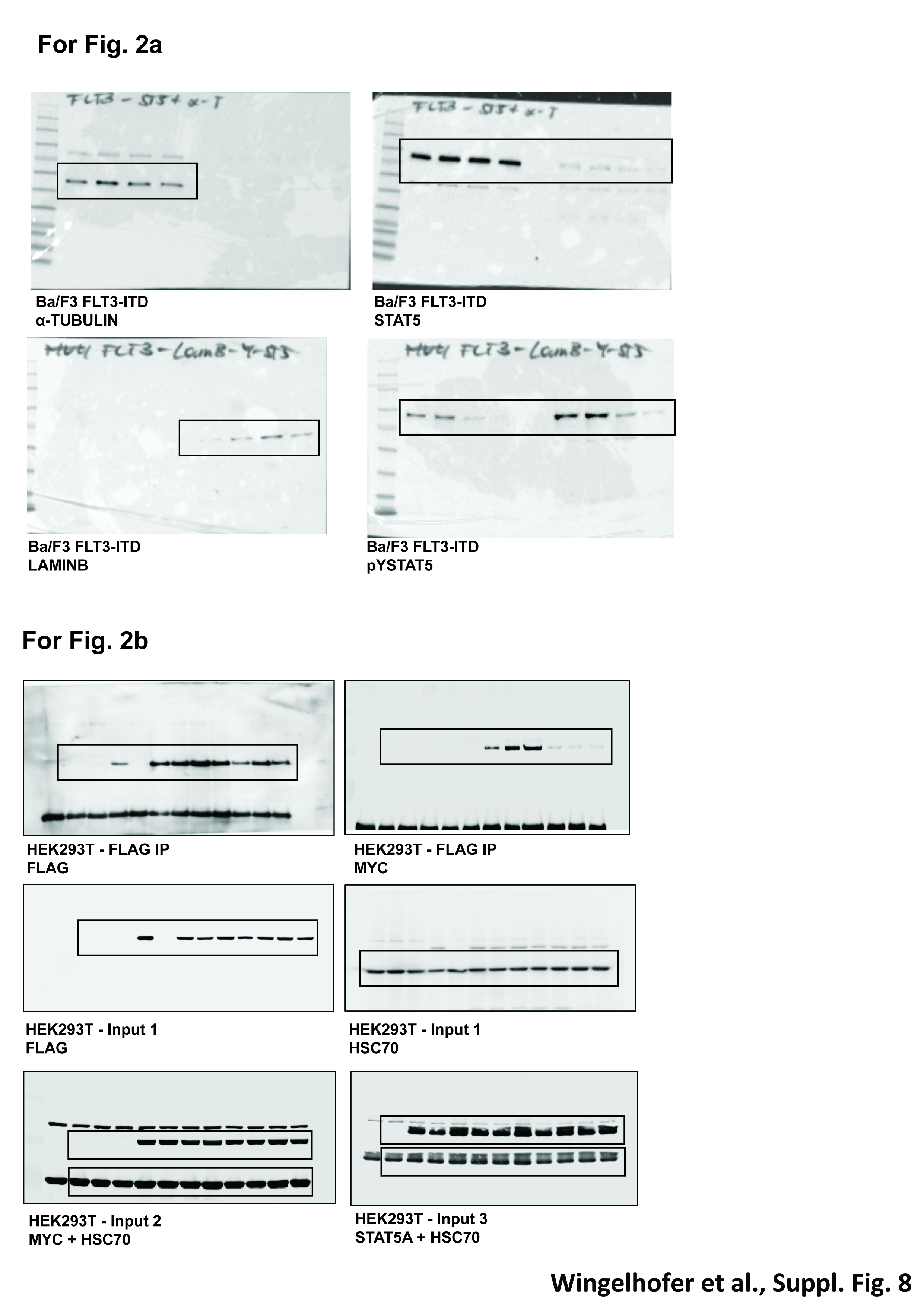

Supplement: Supplementary file 9 — Supplementary Figure 8 [file 41375_2017_5_MOESM9_ESM.tif]

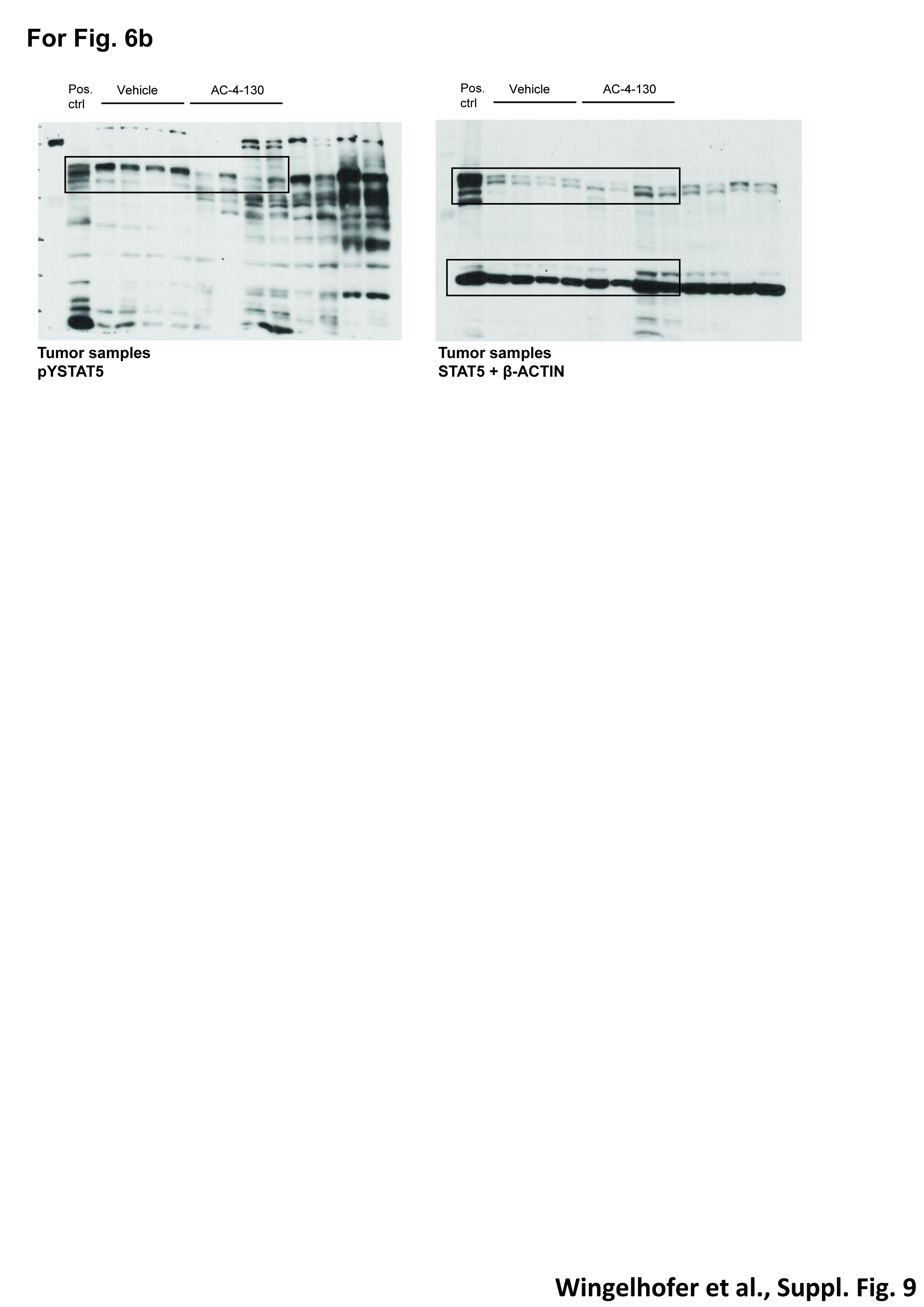

Supplement: Supplementary file 10 — Supplementary Figure 9 [file 41375_2017_5_MOESM10_ESM.tif]

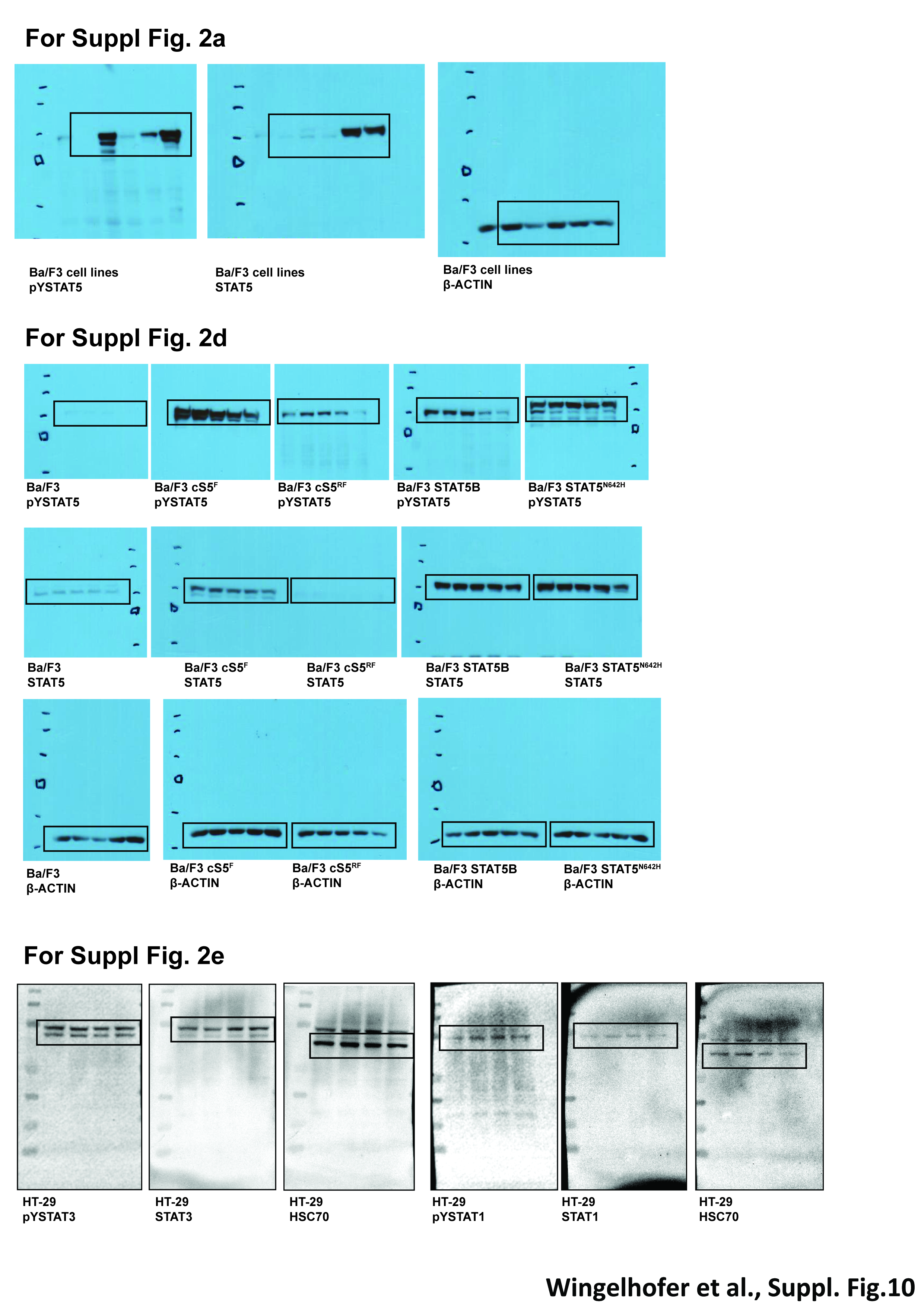

Supplement: Supplementary file 11 — Supplementary Figure 10 [file 41375_2017_5_MOESM11_ESM.tif]

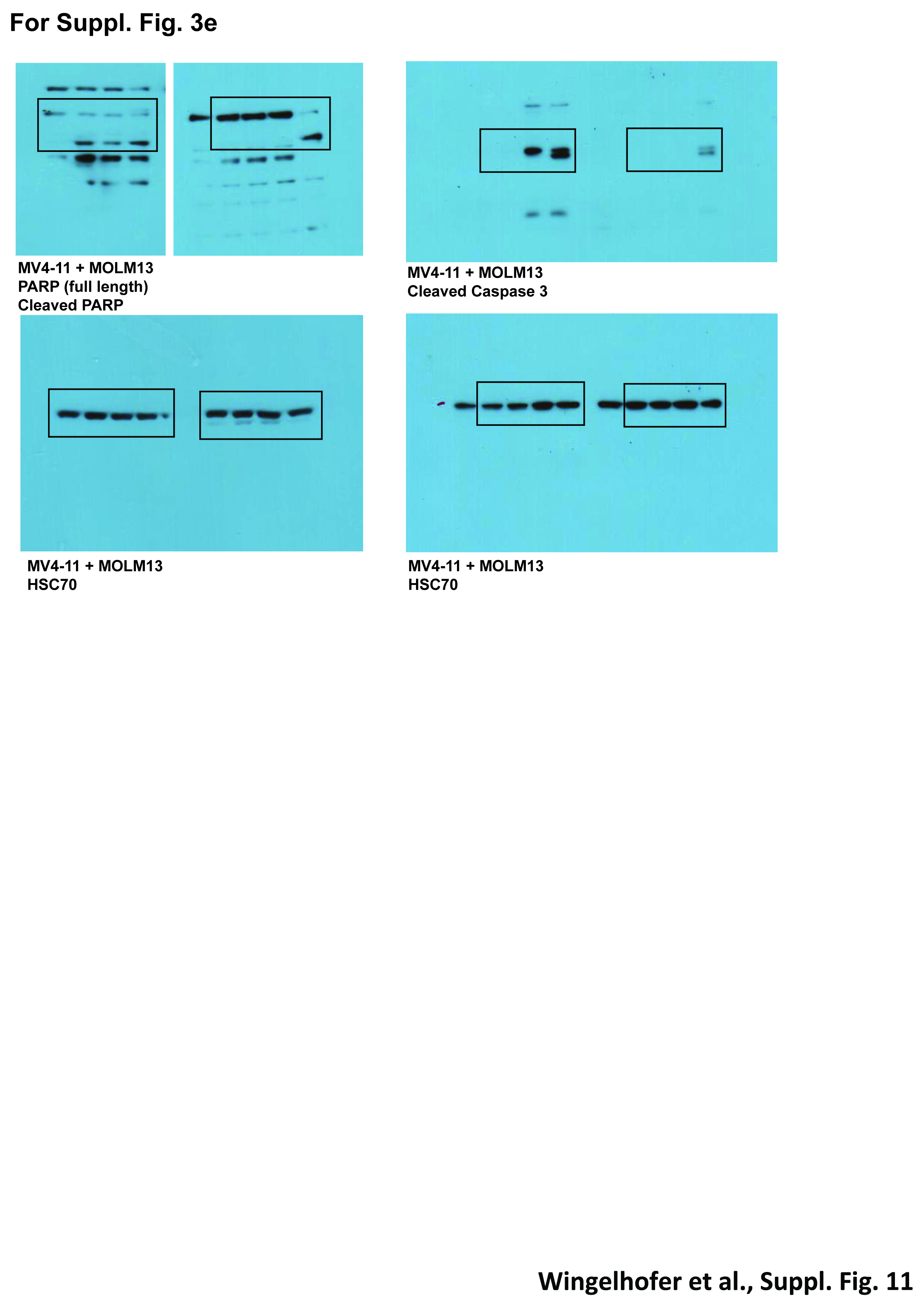

Supplement: Supplementary file 12 — Supplementary Figure 11 [file 41375_2017_5_MOESM12_ESM.tif]
